# Chemical reaction within a compact non-porous crystal containing molecular clusters without the loss of crystallinity†
†Electronic supplementary information (ESI) available: All of the experimental details, crystallographic data collection and refinement statistics, the details of the chemical synthesis, and additional figures and tables. CCDC 1509668–1509670. For ESI and crystallographic data in CIF or other electronic format see DOI: 10.1039/c7sc01041a



**DOI:** 10.1039/c7sc01041a

**Published:** 2017-06-19

**Authors:** Ming Zhang, Tao Yang, Zhenxing Wang, Xiong-Feng Ma, Yuexing Zhang, Samuel M. Greer, Sebastian A. Stoian, Zhong-Wen Ouyang, Hiroyuki Nojiri, Mohamedally Kurmoo, Ming-Hua Zeng

**Affiliations:** a College of Chemistry and Chemical Engineering , Hubei University , Wuhan , 430062 , P. R. China . Email: zmh@hubu.edu.cn; b Department of Chemistry and Pharmaceutical Sciences , Guangxi Normal University , Key Laboratory for the Chemistry and Molecular Engineering of Medicinal Resources , Guilin , 541004 , P. R. China; c Wuhan National High Magnetic Field Center & School of Physics , Huazhong University of Science and Technology , Wuhan , 430074 , P. R. China; d Department of Chemistry and Biochemistry , Florida State University , Tallahassee , Florida 32306 , USA; e National High Magnetic Field Laboratory , Florida State University , Tallahassee , Florida 32310 , USA; f Institute for Materials Research , Tohoku University , Katahira 2-1-1 , Sendai 980-8577 , Japan; g Institut de Chimie de Strasbourg , CNRS-UMR 7177 , Université de Strasbourg , 4 rue Blaise Pascal , 67070 Strasbourg , France . Email: kurmoo@unistra.fr

## Abstract

The very rare occurrence of a gas–solid chemical reaction has been found to take place on a molecule within a compact non-porous crystal without destroying its long-range structural order and retaining similar crystal structures when yellow crystals of FeII4(*mbm*)_4_Cl_4_(MeOH)_4_ were exposed to air to give black [FeIII4(*mbm*)_4_Cl_4_(OH)_4_]·2H_2_O. The latter cannot be synthesised directly. The original cluster underwent an exchange of methanol to hydroxide, an oxidation of Fe(ii) to Fe(iii), a change in stereochemistry and hydration while the packing and space-group remained unaltered.

## Abstract 2

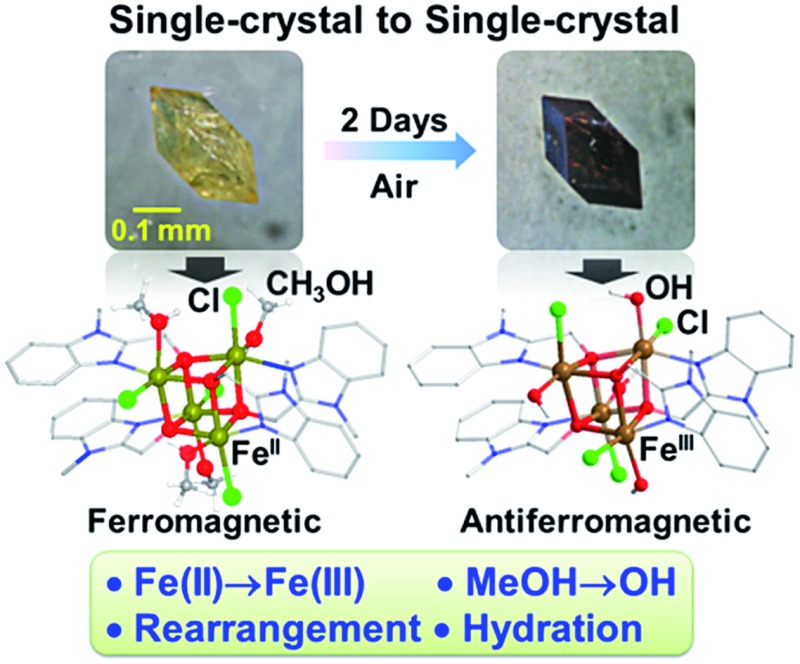
A yellow crystal with {FeII4O_4_} cubes is modified to a black crystal with {FeIII4O_4_} cubes *via* a SC–SC transformation.

## Introduction

One of the requirements for a chemical reaction to take place is that the reactants should be within close proximity for electronic interactions to promote bond breaking and bond formation.^1^ Thus, the reactants have high probability of getting close together when they are in liquid or gas states. Reactions in the solid state require repetitive grinding and mixing before heat treatment, and the crystalline state can then be obtained at high temperatures.^2^ With the exception of oligomerization *via* irradiation or heat treatment, there are rarely reactions that take place in the solid state with retention of the crystallinity.^3^ Gas–solid reactions take place at the surface and invariably destroy the crystalline state of the solid if the structure is non-porous. However, when it is porous, the gaseous reactants can penetrate the structure and modify it without destruction.^4^ Therefore, gas–solid reactions remain exotic, and the advances made in the past twenty years in the field of porous metal–organic frameworks (MOFs) are slowly changing our perceptions and have introduced several new conceptual synthetic approaches leading to good quality crystals.^5–10^ One prominent advance is the post-synthetic modification (PSM) of a crystalline solid without destroying its crystalline state, which has given rise to a new field of single-crystal-to-single-crystal (SC–SC) transformations.^11–18^ Some notable *in situ* advances include (a) desolvation, (b) solvent exchange, (c) coordination at the naked metal sites, and (d) the reaction of the organic moiety. The most remarkable advance is the replacement of the metal centres of a MOF without dissolving the crystals, which has major importance for the development of smart and intelligent materials, as it evidences the process of auto-repairing.^16–18^ All of these advances are possible due to strong connectivity through a combination of covalent and dative bonds within the frameworks and, most importantly, their porous character, which provides space for the reactants to get to the reaction sites. However, when the crystals are non-porous, the compactness of the building units limits the reaction to the surface and, consequently, the crystallinity is destroyed by any form of modification of the crystals through chemical reactions.^19–21^ This is even more likely if the crystals contain molecular units that are held by weak supramolecular interactions.^22^ Two interesting examples have been reported where discrete clusters transform into one-dimensional chains^23^ and layers^24^ and maintain their crystallinity. Notably, Atwood *et al.* reported some non-porous organic solids absorbing various gases without chemical reactions in an SC–SC manner.^25–29^ Two features were involved during the SC–SC transformation of non-porous crystals containing discrete molecules. One is guest transport through the crystal lattice, such as coordinated ligand exchange^20,30,31^ and the addition of H_2_ to a coordinated ligand.^32,33^ The other is charge reorganisation between the metal ion and ligand, such as metal complexes involving tautomerism^34–38^ and hydrogen-atom transfer.^39^ Note that a dimolybdenum molecular pair with a [Mo_2_](μ-OH)_2_[Mo_2_] core undergoes a deprotonation process,^40^ and a dicobalt core is known to serve as an active site for oxygen chemisorption/desorption in a reversible SC–SC transformation.^41^ Therefore, it is considered that chemical reactions that produce molecules within a compact crystalline solid, involving not only guest transport, but also a change in the metal ion charge without destroying the crystal or perturbing the crystalline long-range order, are really very rare. Here, we present a unique gas–solid reaction that can be considered as a different form of rusting, not of iron metal but of a tetranuclear Fe(ii) molecular complex, without destroying its crystalline state, and the gaseous reactants are H_2_O and O_2_ from the atmosphere.

## Results and discussion

**Fig. 1:**
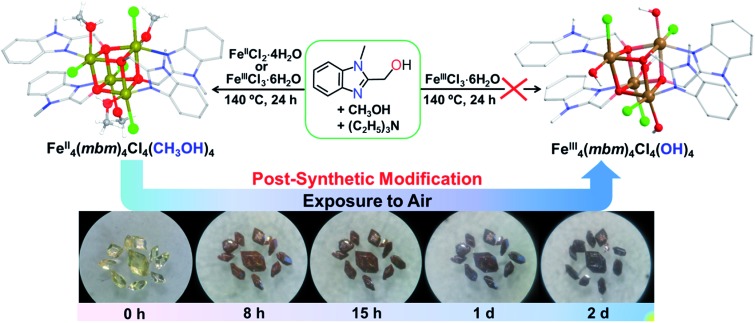
Reaction leading to FeII4(*mbm*)_4_Cl_4_(MeOH)_4_ and its transformation into [FeIII4(*mbm*)_4_Cl_4_(OH)_4_]·2H_2_O. Top: the syntheses adopted. Bottom: time-dependent microscopy photographs of the crystals that were post-synthetically transformed during exposure to air (27 °C, 56% RH).

The yellow crystals of FeII4(*mbm*)_4_Cl_4_(MeOH)_4_ (**1**), obtained *via* a solvothermal reaction of either Fe^II^Cl_2_·4H_2_O or Fe^III^Cl_3_·6H_2_O and (1-methyl-1*H*-benzo[*d*]imidazol-2-yl)methanol (H*mbm*) in methanol and neutralized by triethylamine, slowly converted to black crystals of [FeIII4(*mbm*)_4_Cl_4_(OH)_4_]·2H_2_O (**1-2d**) in air while retaining their crystal morphology (size and shape), and space-group *P*42_1_ *c*, with discrete {Fe_4_O_4_} units. **1-2d** underwent an annealing process in the first 8 days to form **1-8d**, which then lost its crystallinity slowly to form **1-180d** *via* hydration (Fig. 1 and S1†). The results that were obtained are quite unique and reveal a balance of stability as a function of time. Following the determination of the structures from several crystals under non-controllable conditions, we selected three similarly sized virgin yellow crystals of **1** from one batch for a systematic study under ambient conditions (27 °C and 56% RH). The first crystal was used for diffraction data collection within four hours, which reproduced the structure that was found in several other crystals that were studied independently and gave consistent geometrical parameters. The second and third crystals were exposed to air under ambient conditions for 2 days (**1-2d**) and 8 days (**1-8d**), respectively, before collecting the data (Table S1†).

**Fig. 2:**
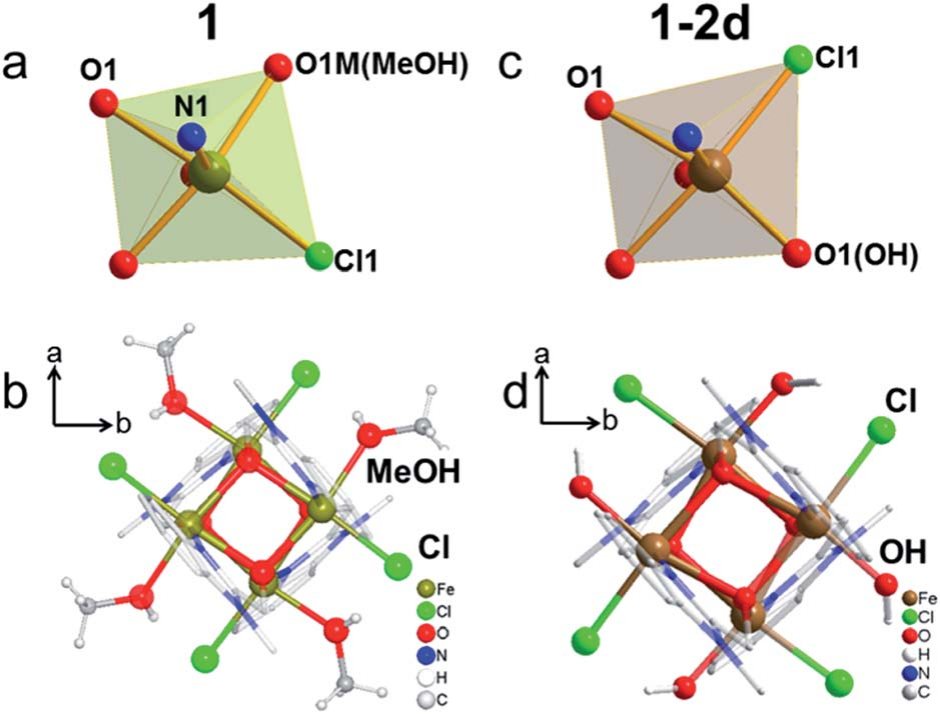
Details of the crystal structures of **1** (left, a and b) and **1-2d** (right, c and d). Top: the coordination modes of Fe; bottom: discrete clusters viewed along the *c*-axis showing the different orientations of the Cl atoms.

The presence of an {Fe_4_O_4_} cubic core is the key feature of the three structures, in which Fe and O atoms occupy alternate corners of a slightly distorted cube (Fig. 2 and Table S2†).^42–45^ They all adopt the non-centrosymmetric space group *P*42_1_ *c*. The asymmetric unit of **1** contains one Fe atom, one *mbm* ligand, one Cl atom and one MeOH molecule. The Fe atom exhibits six-coordination, with one terminal Cl atom in the *cis*-position to one O atom from the terminal MeOH molecule within the same plane of the cube, two O atoms from two *mbm* ligands, and one N atom and one O atom from a chelating *mbm* ligand in an orthogonal plane (Fig. 2a and b). In contrast, the asymmetric unit of **1-2d** contains the same atoms, except that an OH group has replaced the MeOH molecule (Fig. 2c and d). The Fe centre of **1-2d** has the same coordination sphere as that of **1**, but the Cl atom is now in the place of the methanol and the hydroxide is in the position of the Cl atom. The replacement of the neutral methanol by the charged hydroxide increases the oxidation state of Fe from two to three. The Fe(ii)–O distances of **1** lie in the narrow range 2.107–2.181 Å. The Fe(iii)–O distances of **1-2d** were found to lie in the wide range 2.019–2.230 Å. The Fe–O–Fe bridging angles for **1** fall in the narrow range 96.32–99.66°, but again they fall in the wide range 97.64–103.32° for **1-2d**. The distances and angles are comparable to those reported for other {Fe_4_O_4_}^*n*+^ complexes in the literature.^42–45^ **1-2d** and **1-8d** have the same Fe coordination sphere but with slightly different structural parameters, with Fe–O distances of 2.054–2.251 Å and Fe–O–Fe bridging angles of 97.94–103.90°. The Fe–OH distances are 2.019 Å (**1-2d**) and 2.055 Å (**1-8d**). Given the ease of the deprotonation of the terminal hydroxide, leading to the formation of iron oxides,^46,47^ the stability of **1-2d** and **1-8d** is quite remarkable.^48^

To the best of our knowledge, it also has the highest number (4) of terminal hydroxides in a discrete coordination complex. Surprisingly, a discrete cluster containing the fully oxidised FeIII4O_4_ cubane has not been reported, but it exists for a series of octanuclear {Fe_8_} complexes with central FeIII4O_4_.^49,50^ The 24 known discrete {Fe_4_O_4_} cubanes are either divalent Fe(ii) or mixed-valent Fe(ii)/(III) (Table S3†). Therefore, we attempted to synthesize **1-2d** directly using Fe^III^Cl_3_·6H_2_O as the starting material, but all of the attempts have so far resulted in only yellow crystals of **1**. This could be due to poor stability under ambient conditions or the fact that ferric ions tend to connect *via* oxo ligands to form {FeIII4O_6_} layers in solution.^43^ Consequently, **1-2d** and **1-8d** represent the first discrete ferric cubes with terminal hydroxide ligands. The terminal hydroxide ligand is possibly stabilized by a combination of steric hindrance provided by the bulky ligand and the H-bond between the clusters (Fig. S2 and S3†). It is interesting to note that the coordinated Cl atom changes its position during the transformation from **1** to **1-2d** and **1-8d**, while the O and N atoms from *mbm* ligands remain in their original positions.^51^ This suggests that the *mbm* ligand is strongly bonded compared to the methanol. It also implies that an intermediate five-coordinated Fe is formed by the initial departure of the methanol. It is logical to assume that the transformation proceeds gradually from the nucleation sites at the surface to the entire crystal without a loss of crystallinity. However, all of the endeavours to remove MeOH from crystals of **1** under vacuum while retaining its crystallinity have been unsuccessful.

The slow post-synthetic SC–SC modification provides us with an opportunity to track this progressive gas–solid reaction closely. Given the change in colour, our first conclusion is that the compound was being oxidized, which was subsequently confirmed using crystallography. We then performed an experiment to show that water is also required. When **1** was kept in anhydrous methanol, no change in colour was observed after 2 days, but when it was exposed to air, it darkened (Fig. S4†). This suggests that the departed methanol is first replaced by water followed by oxidation leading to Fe^III^ and hydroxide.^52^ Powder X-ray diffraction (PXRD) patterns suggest that the reaction can be stopped by keeping the samples under a nitrogen atmosphere. Time-dependent PXRD patterns with exposure times in air of up to 240 days (Fig. S5†) reveal that the samples diffract well for the first 8 days, but very poorly by 20 days. The results suggest that the crystallinity and long-range order of the cluster in the structure of **1** are maintained up to at least 8 days. Although the diffracting power progressively weakens as a function of time, leading to an almost amorphous solid, the presence of the peak at 2*θ* = 9.0° for **1-180d** indicates the existence of short-range order in the structure up to 180 days after the annealing process.

**Fig. 3:**
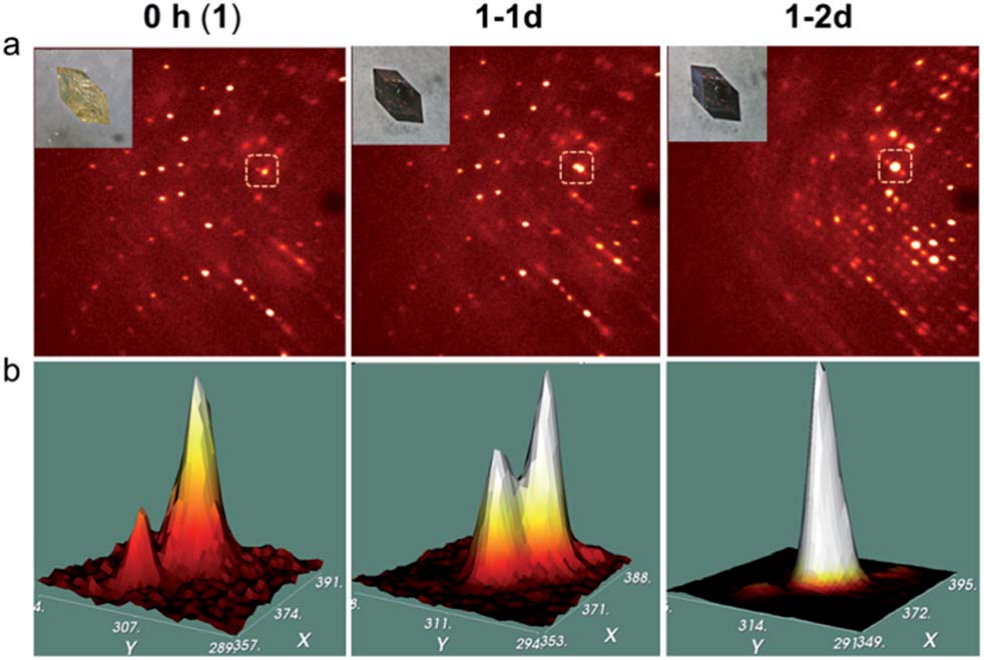
Single crystal X-ray diffraction as a function of exposure time. (a) The diffraction frames and (b) 3D shapes of selected Bragg reflections for **1** after exposure to air for 0 h (left), 24 h (middle) and 48 h (right).

In addition, time dependent crystallography was used on one single crystal. A yellow crystal of **1** was used to collect oscillation frames in air for one orientation as a function of exposure time for up to 50 h (Fig. 3 and S6†) while it was in the enclosure of the Bruker diffractometer under a controlled atmosphere (27 °C; 56% humidity). Zooming in on a selected area of the frames shows a pair of Bragg reflections, one weak and one strong; the intensity ratio changes slowly during 24 h, but by 48 h, only one Bragg reflection is present (Fig. 3b). This suggests the progressive transformation of the phases and the existence of two diffracting lattices from one crystal at the intermediate times without the loss of the crystalline state.^53,54^ We should also note that the presence of the weak peak in the first frame indicates that the crystal has already been partially oxidised during the mounting of the crystal. There are different effects involving SC–SC transformations that have been reported. These are the loss of solvents, the exchange of solvents, the reaction of the organic ligands, and the exchange of the metal. In this context, the SC–SC transformation of **1** to **1-2d** possesses the most chemical changes (Table S4†). Crystallography was used to find both **1** and **1-2d** adopt the same space-group and possess {Fe_4_O_4_} cores with four chemical changes.

An interesting question that follows the above observations is: how do the water and oxygen molecules go through a non-porous crystalline structure? Indeed, this is a very rarely observed process. For the case of the solvation of the crystals containing calixarene, Atwood *et al.* suggested that the guest can be transported through the non-porous solid *via* dynamic van der Waals cooperativity and the expansion of the entire solid.^27,29^ This is not the case here. Therefore, we propose the following plausible process for our case. Because the intercluster interactions between the clusters in the structure of **1** are weak, the MeOH molecule at the surface of the crystal can easily be dissociated, leaving a vacant site at the metal centre for reaction with O_2_ and H_2_O. Since the new oxidised molecular unit is smaller than the original one, the surface is hydrated and the water can move further to neighbouring molecules provoking further reaction, which then propagates through the whole crystal. The expected exothermic energy from the oxidation reaction drives the removal of further MeOH molecules. In contrast, the strong intercluster interactions in the structure of **1-2d** help to maintain the long-range order of the single-crystal lattice. It is also different from the iron rusting case, for example, where the iron crystals are eroded due to the presence of H_2_O and O_2_ (often catalysed by acidic gases) to form iron-oxide crystals at the surface.

**Fig. 4:**
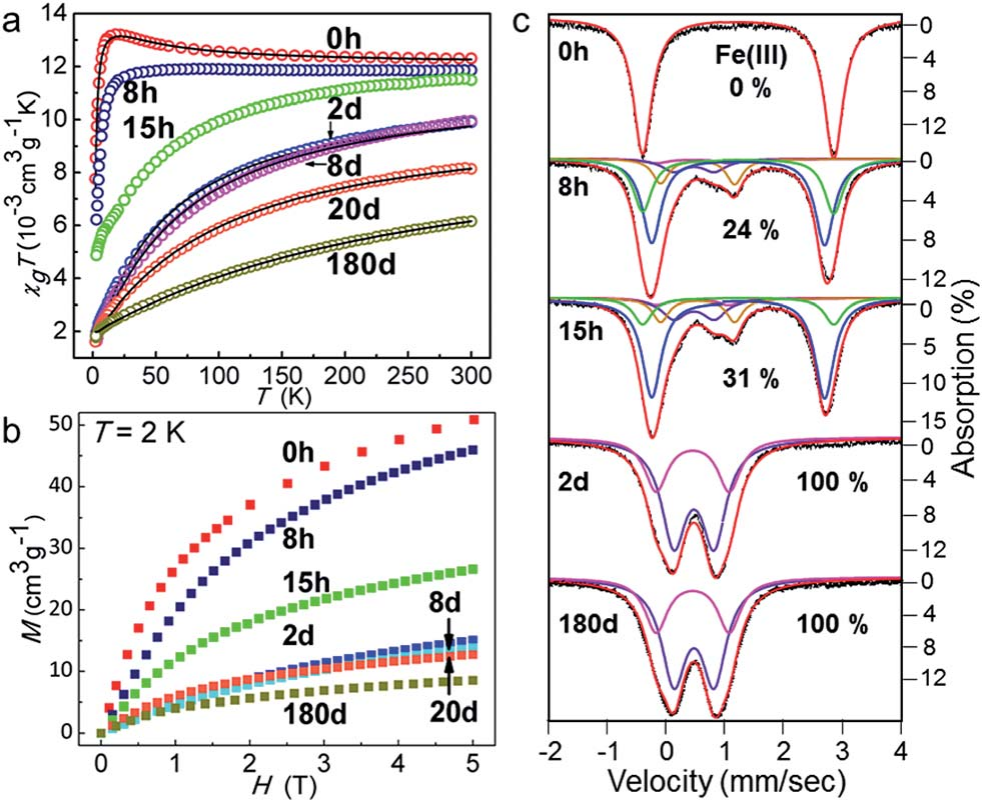
(a) The temperature dependence of *χ*_g_ *T* in 1 kOe for the samples that were exposed to air for different periods of time. The solid lines represent the theoretical fits using the parameters given in the ESI;† (b) their isothermal magnetisation at 2 K; and (c) zero-field ^57^Fe Mössbauer spectra at 80 K for the fresh sample (**1**), and samples of **1-8h**, **1-15h**, **1-2d**, and **1-180d** that were exposed to air. The simulations shown in red correspond to the sum of all of the components.

The oxidation of Fe(ii) to Fe(iii) introduces a change in the spin and orbital states of the magnetic ions. Therefore, we have followed the changes in the magnetic properties using a SQUID magnetometer, and HF-EPR and Mössbauer spectroscopy (further details are given in the ESI†). The high temperature susceptibility data reveal a change from dominant ferromagnetic (*θ* = +2.8(2) K for **1**, from Curie–Weiss law fitting) to strong antiferromagnetic exchange (*θ* = –53.0(2) K for **1-2d**) with time (Fig. 4a and Table S5†). The results from the fitting of the magnetic data (Fig. S7†) correlate well with those observed for related compounds and those calculated using DFT (Table S6†).^42–45,49,50^ From the HF-EPR spectra at low temperatures and different frequencies, *g*-values of 1.49, 2.92, 3.61 and 5.50 and three energy gaps of 27, 46, and 190 GHz were extracted (Fig. S8†), which confirm the ZFS of the Fe(ii) atom in **1**. These gaps are in good agreement with the values of *D* (14.3(1) cm^–1^) and *E* (2.1(1) cm^–1^) obtained from modelling the high temperature data.^55–59^ **1-2d**, **1-8d** and **1-180d** only have two resonances that correspond to *g*-values of 2.19 and 2.11 for **1-2d**, 2.08 and 2.04 for **1-8d**, and 2.09 and 1.99 for **1-180d**, but they have small energy gaps of ∼20 GHz that are consistent with those of singlet Fe(iii) ions.^41^ Due to the increasing AF exchange energy with time, the isothermal magnetization is harder to saturate with a field (Fig. 4b). Moreover, Mössbauer spectroscopy also confirmed that all of the Fe(ii) ions in the molecular cluster completely oxidised to Fe(iii), and gives a more accurate proportion of the different valences (Fig. 4c and S9–S10 and Tables S8–S10†). The temperature dependence of the ac-susceptibility for **1** and **1-2d** indicates there is no single-molecule magnetic behaviour above 1.8 K (Fig. S11†).

## Conclusions

In summary, the progressive post-synthetic transformation of a yellow ferrous cubane cluster, FeII4(*mbm*)_4_Cl_4_(MeOH)_4_, into its dark ferric congener, [FeIII4(*mbm*)_4_(OH)_4_Cl_4_]·2H_2_O, as a function of exposure to air has been observed and explored using crystallography, magnetometry, and HF-EPR and Mössbauer spectroscopy. This unique single-crystal-to-single-crystal transformation prevails up to 8 h as the Fe^II^ ions are oxidised to Fe^III^ ions, but the crystallinity degrades slowly afterwards due to disorder induced by water intake. Although SC–SC transformations involving non-porous molecular materials have been reported, to the best of our knowledge, no material has such abundant guest transport through the crystal lattice, with dioxygen entering and methanol departing. In particular, four chemical changes were noted: (a) the replacement of the methanol by the hydroxide (Fig. S12†), (b) a coordination site swap of the chlorine atom within the Fe octahedron, (c) the oxidation of Fe and (d) hydration. The consequence of these changes is reflected in the SQUID magnetometry results, where a progressive change from ferromagnetic coupling with considerable single-ion anisotropy for the virgin yellow crystals to strongly antiferromagnetic coupling and weak anisotropy for the oxidised dark crystals is observed. Mössbauer spectra confirm the complete oxidation of Fe^II^ to Fe^III^ and gave more accurate proportions of the two valences at different times. This astonishing retention of the crystalline state through the three chemical changes on a molecule can be regarded as a gas–solid state reaction.

## Experimental

All of the reagents were obtained from commercial sources and used without further purification. Elemental analyses for C, H, and N were performed using a Vario Micro Cube. Infrared spectra were recorded *via* transmission through KBr pellets containing *ca*. 0.5% of the compounds using a PE Spectrum FT-IR spectrometer (400–4000 cm^–1^). The X-ray powder diffraction patterns were measured at 293 K using a Bruker APEX-II CCD diffractometer (Mo Kα, *λ* = 0.71073 Å). The magnetization data for the polycrystalline samples were measured in the temperature range 2–300 K and the field of ±50 kOe using a Quantum Design MPMS XL-7 SQUID magnetometer. High frequency electron paramagnetic resonance (HF-EPR) spectra were recorded on locally developed instruments at the Wuhan National High Magnetic Field Center, China and the Institute of Materials Research, Sendai, Japan. The Nuclear Gamma Resonance (Mössbauer) spectra were recorded in zero-field at 80 K using a constant acceleration spectrometer. The source consisted of ∼8 mCi ^57^Co dispersed in rhodium foil. This instrument was fitted with a Janis cryostat that was cooled using liquid nitrogen. The powder samples were contained in custom-made plastic containers. Spectral simulations were performed using WMOSS software (WEB research, Edina, MN). Isomer shifts are quoted relative to the centroid of the iron metal spectrum that was recorded at room-temperature.

### Synthesis

A mixture of FeCl_2_·4H_2_O (1.0 mmol, 198 mg), H*mbm* (3.0 mmol, 486 mg), triethylamine (0.1 mL) and methanol (8 mL) was sealed in a 15 mL Teflon-lined steel autoclave and heated at 140 °C for one day. After the autoclave was cooled to room temperature at a rate of 10 °C min^–1^, light yellow rhombic crystals of **1** were obtained. Yield, 220 mg, 20% (based on Fe). Anal. for **1**: calcd for [FeII4(C_9_H_9_N_2_O)_4_Cl_4_(CH_3_OH)_4_]: C 42.21, H 4.61, and N 9.85; found (%): C 41.54, H 4.63, and N 9.80. Using the alternative starting material FeCl_3_·6H_2_O (0.33 mmol, 73 mg) and H*mbm* (1.0 mmol, 162 mg), triethylamine (0.1 mL) and methanol (8 mL) under similar conditions resulted in light yellow rhombic crystals of **1** (75 mg, yield 30%). Fe(iii) is reduced to Fe(ii) under solvothermal conditions in the presence of methanol. **1-2d**, **1-8d** and **1-180d** were obtained by exposing crystals of **1** to air at ambient temperature for 2, 8 and 180 days, respectively (Fig. S1†). The reaction rate somehow varied upon the change of ambient temperature and humidity. The rate of this blackening appears to have been faster for the smaller crystals. The final phase of the black crystals was then identified using X-ray diffraction, while the solvent was confirmed using TG-IR spectroscopy and EA. Calcd for [FeIII4(C_9_H_9_N_2_O)_4_Cl_4_(OH)_4_]·2H_2_O (**1-2d**): C 38.81, H 3.98, and N 10.06; found (%): C 38.86, H 3.95, and N 10.05. Calcd for [FeIII4(C_9_H_9_N_2_O)_4_Cl_4_(OH)_4_]·2H_2_O (**1-8d**): C 38.81, H 3.98, and N 10.06; found (%): C 38.89, H 3.99, and N 10.03. Calcd for [FeIII4(C_9_H_9_N_2_O)_4_Cl_4_(OH)_4_]·4H_2_O (**1-180d**): C 37.60, H 4.21, and N 9.24; found (%): C 37.51, H 3.92, and N 9.79. Supplementary crystallographic data can be found in the ESI.† The CCDC reference numbers are ; 1509668 (**1**), ; 1509669 (**1-2d**) and ; 1509670 (**1-8d**). Crystal data for (**1**): *P*42_1_c, *a* = 11.148(2) Å, *c* = 19.893(4) Å, *V* = 2472.3(10) Å^3^, *M*_r_ = 1138.09, *D*_c_ = 1.529 g cm^–3^, *Z* = 2, *R*_1_ = 0.0322 (*I* > 2*σ*(*I*)), *wR*_2_ = 0.0819 (all data), *S* = 1.067, and Flack parameter = –0.013(6). (**1-2d**): *P*42_1_c, *a* = 10.960(1) Å, *c* = 18.392(1) Å, *V* = 2209.3(10) Å^3^, *M*_r_ = 1109.96, *D*_c_ = 1.669 g cm^–3^, *Z* = 2, *R*_1_ = 0.0479 (*I* > 2*σ*(*I*)), *wR*_2_ = 0.1243 (all data), *S* = 1.100, and Flack parameter = 0.03(4). (**1-8d**): *P*42_1_c, *a* = 11.084(1) Å, *c* = 18.299(1) Å, *V* = 2248.2(7) Å^3^, *M*_r_ = 1109.96, *D*_c_ = 1.640 g cm^–3^, *Z* = 2, *R*_1_ = 0.0484 (*I* > 2σ(*I*)), *wR*_2_ = 0.1505 (all data), *S* = 1.145, and Flack parameter = 0.002(14).
