# Supplementary material for: Chemical reaction within a compact non-porous crystal containing molecular clusters without the loss of crystallinity
Source: Chem Sci. 2017 Jun 19;8(8):5356–61. doi: 10.1039/c7sc01041a (PMC5609145; doi:10.1039/c7sc01041a)
Supplement: Supplementary file 1 [file SC-008-C7SC01041A-s001.pdf]

Electronic Supplementary Material (ESI) for Chemical Science.
This journal is © The Royal Society of Chemistry 2017

Supporting Information

Chemical Reaction within a Compact Non-Porous Crystal Containing Molecular Clusters without Lost of Crystallinity

Ming Zhang[⊥], Tao Yang[⊥], Zhenxing Wang[⊥], Xiong-Feng Ma, Yuexing Zhang, Samuel M. Greer, Sebastian A. Stoian, Zhong-Wen Ouyang, Hiroyuki Nojiri, Mohamedally Kurmoo* and Ming-Hua Zeng*

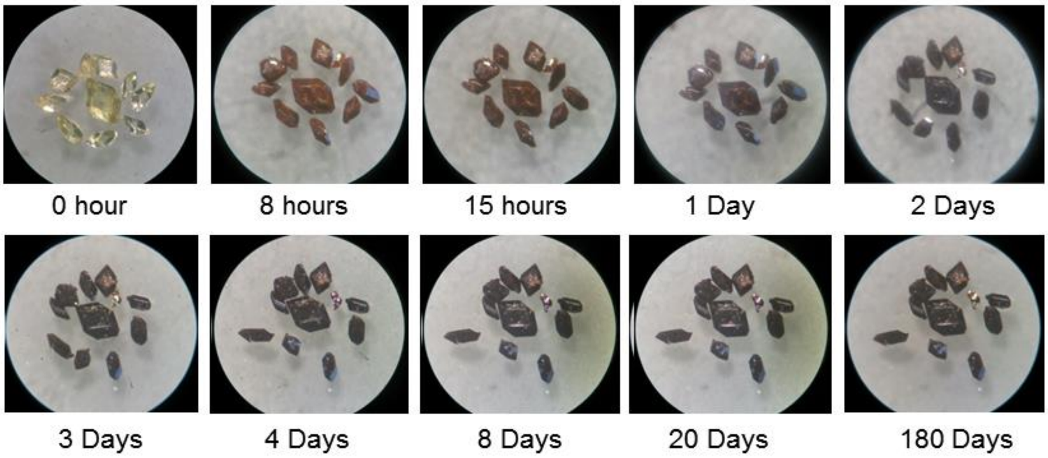

(a)

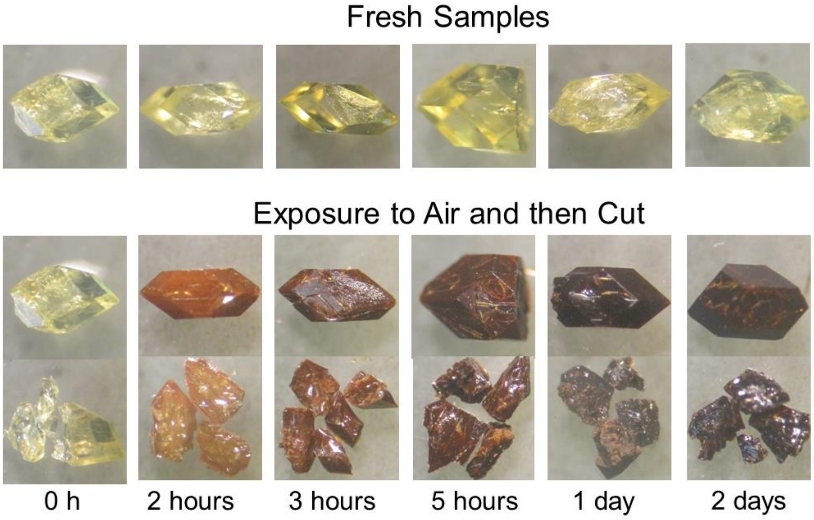

(b)

Figure S1. (a) Time-dependent optical photography of crystals of **1** exposed to air (27 ℃, 56% RH). In the first 2 days, the colour of crystals gradually changes from light yellow to opaque black. After two days, the colour of the surface of crystals does not change. (b) Time-dependent optical photography of crystals of **1** exposed to air (31 ℃, 64% RH). The surface colour is darker than the interior of the crystals. This result further confirmed that the oxidation procedure start from the surface then moves slowly to the inside of the crystal.

Crystal structure determinations.

(1) Selected single crystals of suitable sizes were mounted on glass fibres for diffraction data collections. Diffraction data of **1**, **1-2d** and **1-8d** were collected on a Bruker APEX-II CCD diffractometer employing graphite-monochromated Mo-Kα radiation (λ = 0.71073 Å). The structures were solved using ShelXS (Sheldrick, G.M. *Acta Cryst. A.* **2008**, *64*, 112-122.) The structural solutions were found by Direct Methods and refined using the ShelXL package by least squares minimization. The structures were each refined as a two-component inversion twin. In the structure of **1-2d** and **1-8d**, O1w atoms located at special positions and their site occupation factors were 0.5. The final structures were examined using the Addsym subroutine of PLATON to assure that no additional symmetry could be applied to the models (Spek, A. L. *Acta Cryst.* **2009**, *D65*, 148-155). All non-hydrogen atoms were refined with anisotropic thermal factors.

Table S1. Crystal and structure refinement data for **1**, **1-2d** and **1-8d**.

	**1**	**1-2d**	**1-8d**
Empirical formula	$C_{40}H_{52}Cl_4Fe_4N_8O_8$	$C_{36}H_{40}Cl_4Fe_4N_8O_{10}$	$C_{36}H_{40}Cl_4Fe_4N_8O_{10}$
Formula weight	1138.09	1109.96	1109.96
T (K)	298(2)	298(2)	298(2)
Crystal System	Tetragonal	Tetragonal	Tetragonal
Space group	P-42$_1c$	P-42$_1c$	P-42$_1c$
a / Å	11.148(2)	10.960(1)	11.084(1)
b / Å	11.148(2)	10.960(1)	11.084(1)
c / Å	19.893(4)	18.392(1)	18.299(3)
V / Å^3	2472.3(10)	2209.32 (10)	2248.2(7)
Z	2	2	2
F(000)	1168	1128	1168
D_{calcd} (g/cm^3)	1.529	1.669	1.640
μ(mm^{-1})	1.422	1.593	1.565
θ range (^{o})	2.047-31.069	2.163-29.958	2.148-26.832
Reflection coll./unique	37744 / 3821	30350 / 3142	18840 / 2411
R_{int}	0.1078	0.0596	0.0400
GOF	1.067	1.100	1.145
R_1^a(I>2σ(I))	0.0322	0.0479	0.0484
wR_2^b (all data)	0.0819	0.1243	0.1505
Flack	-0.013(6)	0.03(4)	0.002(14)
$\Delta\rho_{max}$, $\Delta\rho_{min}$, (e. Å^{-3})	0.609, -0.547	0.626, -0.435	0.610, -0.501
U_{ij} parameter of Cl	0.03512	0.04838	0.06022
	0.05372	0.05336	0.06525
	0.06684	0.05565	0.06065
	-0.00178	-0.00618	-0.00726
	0.00385	0.00494	0.00512
	-0.01285	0.01336	0.01513
U_{ij} parameter of O	(methanol)	(hydroxide)	(hydroxide)
	0.04345	0.04636	0.05259
	0.05892	0.05995	0.06408

	0.08447	0.06566	0.07502
	0.02022	0.00231	0.01019
	0.01580	0.00620	0.00192
	0.01636	0.01293	0.00804

[a] $R_1=\Sigma||F_o|-|F_c||/\Sigma|F_o|$, [b] $wR_2=[\Sigma w(F_o^2-F_c^2)^2/\Sigma w(F_o^2)^2]^{1/2}$.

Table S2. Selected Bond Lengths (Å) and Angles (°) for **1**, **1-2d** and **1-8d**.

	**1**		
Fe(1)—O(1)#1	2.107 (2)	Fe(1)—O(1)#2	2.181 (2)
Fe(1)—N(1)	2.141 (2)	Fe(1)—O(1)#3	2.107 (2)
Fe(1)—O(1)	2.176 (3)	Fe(1)—Cl(1)	2.405 (1)
Fe(1)—O(1M)	2.178 (2)	Fe(1) #2—O(1)	2.181 (2)
O(1)#1—Fe(1)—N(1)	158.16(8)	O(1M)—Fe(1)—O(1)#2	167.10(8)
O(1)#1—Fe(1)—O(1)	82.81(7)	O(1)#1—Fe(1)—Cl(1)	99.61(5)
N(1)—Fe(1)—O(1)	75.90(7)	N(1)—Fe(1)—Cl(1)	102.01(6)
O(1)#1—Fe(1)—O(1M)	87.61(9)	O(1)—Fe(1)—Cl(1)	174.67(5)
N(1)—Fe(1)—O(1M)	87.62(10)	O(1M)—Fe(1)—Cl(1)	94.67(8)
O(1)—Fe(1)—O(1M)	90.16(9)	O(1)#2—Fe(1)—Cl(1)	95.33(5)
O(1)#1—Fe(1)—O(1)#2	82.70(7)	Fe(1)#3—O(1)—Fe(1)	96.46(6)
N(1)—Fe(1)—O(1)#2	98.24(7)	Fe(1)#3—O(1)—Fe(1)#2	96.32(7)
O(1)—Fe(1)—O(1)#2	80.22(7)	Fe(1)—O(1)—Fe(1)#2	99.66(7)
	**1-2d**		
Fe(1)—O(2)	2.019(5)	Fe(1)—O(1)#2	2.230(4)
Fe(1)—O(1)#1	2.070(4)	Fe(1)—Cl(1)	2.369(2)
Fe(1)—N(1)	2.097(4)	O(1)—Fe(1)#3	2.070(4)
Fe(1)—O(1)	2.162(3)	O(1)—Fe(1)#2	2.230(4)
O(2)—Fe(1)—O(1)#1	101.1(2)	O(1)—Fe(1)—O(1)#2	76.45(14)
O(2)—Fe(1)—N(1)	102.13(19)	O(2)—Fe(1)—Cl(1)	95.66(14)
O(1)#1—Fe(1)—N(1)	156.09(16)	O(1)#1—Fe(1)—Cl(1)	91.99(10)
O(2)—Fe(1)—O(1)	166.33(17)	N(1)—Fe(1)—Cl(1)	91.33(13)
O(1)#1—Fe(1)—O(1)	80.38(16)	O(1)—Fe(1)—Cl(1)	97.87(11)
N(1)—Fe(1)—O(1)	75.71(15)	O(1)#2—Fe(1)—Cl(1)	169.81(11)
O(2)—Fe(1)—O(1)#2	90.44(16)	Fe(1)#3—O(1)—Fe(1)	99.80(15)
O(1)#1—Fe(1)—O(1)#2	78.80(15)	Fe(1)#3—O(1)—Fe(1)#2	97.64(15)
N(1)—Fe(1)—O(1)#2	95.37(15)	Fe(1)—O(1)—Fe(1)#2	103.32(14)
	**1-8d**		
Fe(1)—O(2)	2.054(7)	Fe(1)—O(1)#2	2.251(5)
Fe(1)—O(1)#1	2.063(5)	Fe(1)—Cl(1)	2.381(2)
Fe(1)—N(1)	2.092(6)	O(1)—Fe(1)#3	2.063(5)
Fe(1)—O(1)	2.179(5)	O(1)—Fe(1)#2	2.251(5)
O(2)—Fe(1)—O(1)#1	101.4(2)	O(1)—Fe(1)—O(1)#2	75.86(19)
O(2)—Fe(1)—N(1)	102.4(2)	O(2)—Fe(1)—Cl(1)	96.00(18)
O(1)#1—Fe(1)—N(1)	155.3(2)	O(1)#1—Fe(1)—Cl(1)	92.55(14)
O(2)—Fe(1)—O(1)	165.7(2)	N(1)—Fe(1)—Cl(1)	91.38(17)
O(1)#1—Fe(1)—O(1)	79.9(2)	O(1)—Fe(1)—Cl(1)	98.21(15)
N(1)—Fe(1)—O(1)	75.44(19)	O(1)#2—Fe(1)—Cl(1)	169.69(13)
O(2)—Fe(1)—O(1)#2	90.4(2)	Fe(1)#3—O(1)—Fe(1)	100.26(19)
O(1)#1—Fe(1)—O(1)#2	78.20(19)	Fe(1)#3—O(1)—Fe(1)#2	97.94(19)
N(1)—Fe(1)—O(1)#2	95.19(19)	Fe(1)—O(1)—Fe(1)#2	103.90(19)

Symmetry transformations used to generate equivalent atoms: #1 –y –1, x, −z−2; #2 –x –1, –y –1, z; #3 y, –x –1, −z−2.

Table S3. Literature survey for structures containing discrete Fe_4O_4-core. (CCDC database, May 2016)

No.	Ligand	Complexes	Ref.
**Fe(II)- or Fe(II)/(III)-based Fe_4O_4 core**			
**1**	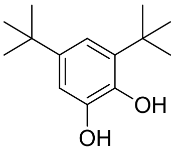	$[Fe^{II}_4(3,5\text{-t-BUz}C_6H_2O_2)_4(py)_6]$	1
**2**	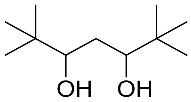	$[Fe^{II}(OMe)(MeOH)(DPM)]_4$	2
**3**	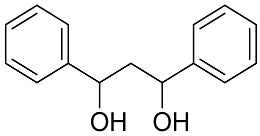	$[Fe^{II}(OMe)(MeOH)(DBM)]_4$	2
**4**	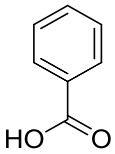	$[Fe^{II}_3Fe^{III}(OMe)_5(MeOH)_3(OBz)_4]$	2
**5**	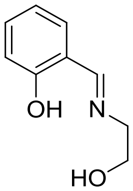	$[Fe^{II}(OMe)(MeOH)(dpm)]_4$	3
**6**	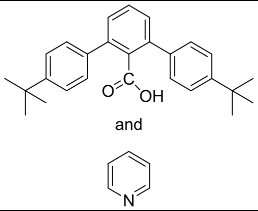	$[Fe^{II}_4(\mu\text{-}OH)_4(\mu\text{-}O_2CAr^{4\text{-tBuPh}})_2(\mu\text{-}OTf)_2(C_5H_5N)_4]$	4
**7**	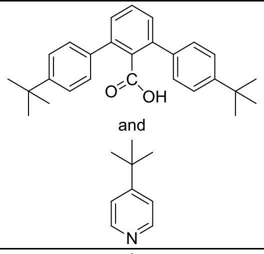	$[Fe^{II}_4(\mu\text{-}OH)_4(\mu\text{-}O_2CAr^{4\text{-tBuPh}})_2(\mu\text{-}OTf)_2(4\text{-}^tBuC_5H_4N)_4]$	4
**8**	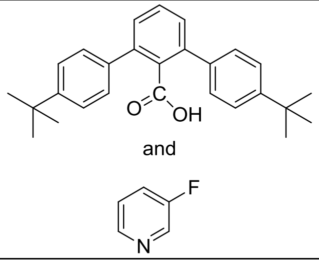	$[Fe^{II}_4(\mu\text{-}OH)_4(\mu\text{-}O_2CAr^{4\text{-tBuPh}})_2(\mu\text{-}OTf)_2(3\text{-}FC_5H_4N)_4]$	4

**9**	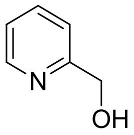 and 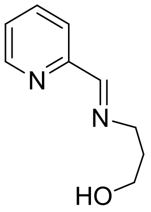	$[Fe^{II}_4(pypentO)(pym)_3(Oac)(NCS)_3]\cdot 1.5EtOH$	5
**10**	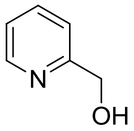 and 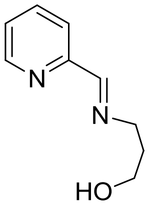	$[Fe^{II}_4(pypentO)(pym)(Oac)_2(NCS)_2(MeO)_2(H_2O)]\cdot H_2O$	5
**11**	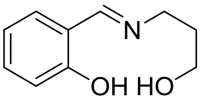	$[Fe^{II}_4(sap)_4(MeOH)_4]\cdot 2H_2O$	6
**12**	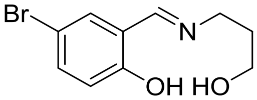	$[Fe^{II}_4(5\text{-}Br\text{-}sap)_4\text{-}(MeOH)_4]$	6
**13**	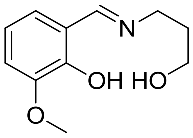	$[Fe^{II}_4(3\text{-}MeO\text{-}sap)_4(MeOH)_4]$	6
**14**	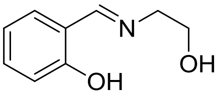	$[Fe^{II}_4(sae)_4(MeOH)_4]$	6
**15**	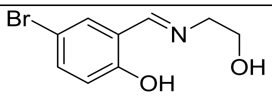	$[Fe^{II}_4(5\text{-}Br\text{-}sae)_4(MeOH)_4]\cdot MeOH$	6
**16**	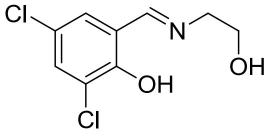	$[Fe^{II}_4(3,5\text{-}Cl_2\text{-}sae)_4(MeOH)_4]$	6
**17**	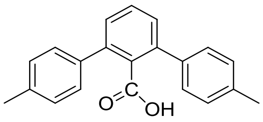	$[Fe^{II}_4(\mu\text{-}OMe)_4(O_2CAr^{Tol})_4(MeOH)_6]\cdot MeOH$	7

**18**	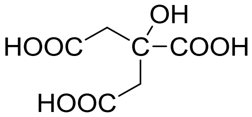	$[Fe^{II}_4(cit)_4][C(NH_2)_3]_8 \cdot 8H_2O$	8
**19**	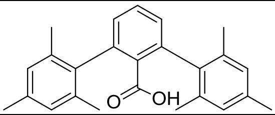	$Fe^{II}_4(Mes_2ArCO_2)_4(NO)_4(\mu_3\text{-}OH)_4$	9
**20**	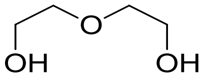	$Fe^{II}_4Cl_4(OCH_2CH_2OCH_2CH_2OH)_4$	10
**21**	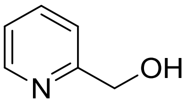	$[Fe^{II}(hmp)(EtOH)Cl]_4$	11
**22**	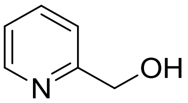	$[Fe^{II}(hmp)(MeOH)Cl]_4$	11
**23**	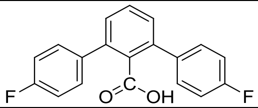	$[Fe^{II}_4(\mu_3\text{-}OMe)_4(O_2CAr_4^{F\text{-}PhI})_4(HOMe)_8] \cdot 0.5HOMe$	12
**24**	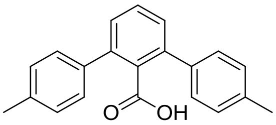	$[Fe^{II}_4(\mu_3\text{-}OMe)_4(O_2CAr_4^{Tol})_4(HOMe)_6] \cdot HOMe$	12
**25**	**1**	$[Fe^{II}_4(C_9H_9N_2O)_4(Cl)_4(CH_3OH)_4]$	This work
	**Fe(III)-based Fe_4O_4 core or discrete cluster**		
**1**	**1-2d, 1-8d**	$[Fe^{III}_4(C_9H_9N_2O)_4(Cl)_4(OH)_4] \cdot 2H_2O$	This work

References

(1) Shoner, S. C.; Power, P. P. *Inorg. Chem.* **1992**, *31*, 1001-1010.

(2) Taft, K. L.; Caneschi, A.; Pence, L. E.; Delfs, C. D.; Papaefthymiou, G. C.; Lippard, S. J. *J. Am. Chem. Soc.* **1993**, *115*, 11753-11766

(3) Oshio, H.; Hoshino, N.; Ito, T. *J. Am. Chem. Soc.* **2000**, *122*, 12602-12603.

(4) Lee, D.; Sorace, L.; Caneschi, A.; Lippard, S. J. *Inorg. Chem.* **2001**, *40*, 6774-6781.

(5) Clemente-Juan, J. M.; Mackiewicz, C.; Verelst, M.; Dahan, F.; Bousseksou, A.; Sanakis, Y.; Tuchagues, J.-P. *Inorg. Chem.* **2002**, *41*, 1478-1491.

(6) Abrahams, B. F.; Hudson, T. A.; Robson, R. *J. Am. Chem. Soc.* **2004**, *126*, 8805-8812.

(7) Yoon, S.; Lippard, S. J. *J. Am. Chem. Soc.* **2005**, *127*, 8386-8397.

(8) Hudson, T. A.; Berry, K. J.; Moubaraki, B.; Murray, K. S.; Robson, R. *Inorg. Chem.* **2006**, *45*, 3549-3556.

(9) Klein, D. P.; Young, Jr., V. G.; Tolman, W. B.; Que, Jr., L. *Inorg. Chem.* **2006**, *45*, 8006-8008.

(10) Diewald, S.; Lan, Y.; Clérac, R.; Powell, A.-K.; Feldmann, C. *Z. Anorg. Allg. Chem.* **2008**, 634, 1880-1886.

(11) Piga, F.; Moro, F.; Krivokapic, I.; Blake, A. J.; Edge, R.; McInnes, E. J. L.; Evans, D. J.; McMastera, J.; van Slageren, J. *Chem. Commun.* **2012**, *48*, 2430-2432.

(12) Ponomaryov, A. N.; Kim, N.; Hwang, J.; Nojiri, H.; van Tol, J.; Ozarowski, A.; Park, J.; Jang, Z.; Suh, B.; Yoon, S.; Choi, K.-Y. *Chem. Asian J.* **2013**, *8*, 1152-1159.

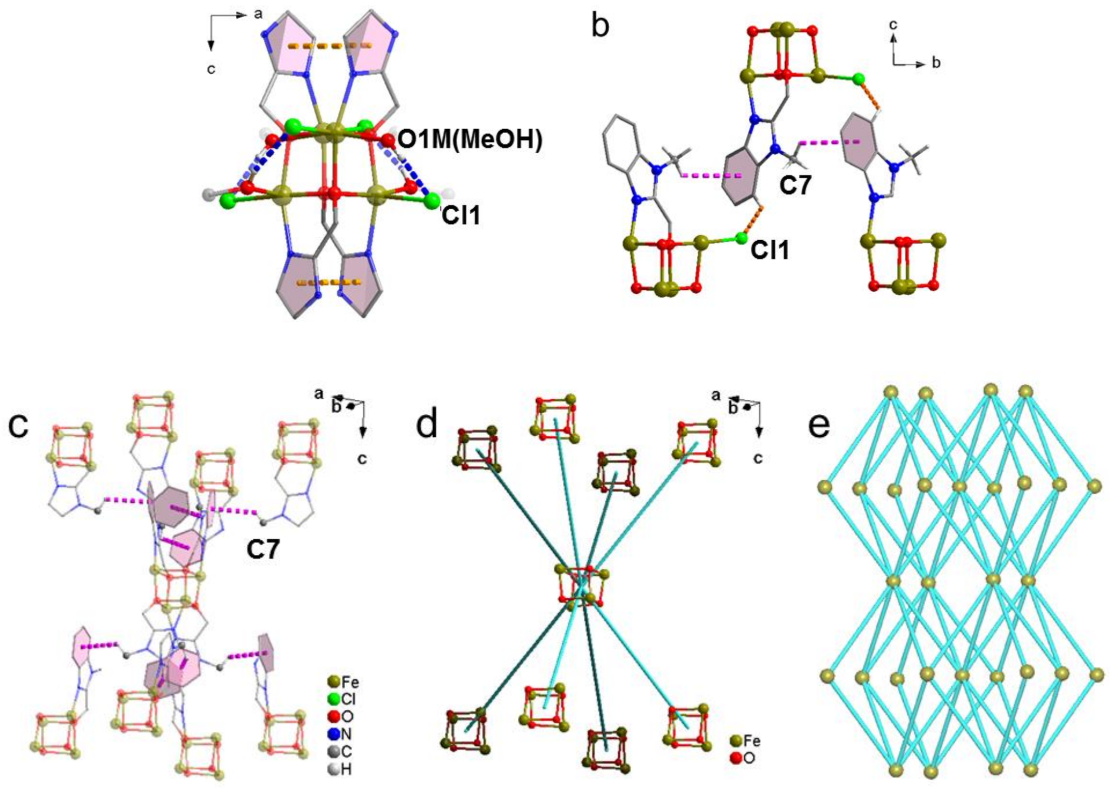

Figure S2. Supramolecular interactions within the crystal structure of **1**. (a) O(MeOH)–H···Cl H-bond (blue) and π···π intracluster (light orange); (b) C–H···π (pink) interactions. (c) C(Me)–H···π intercluster (pink dotted line) interaction in the structure of **1**; (d) 8-connected intercluster through C(Me)–H···π interaction (cyan solid line, 12.69 Å). (e) Uninodal 8-connected **bcu** net of **1.**

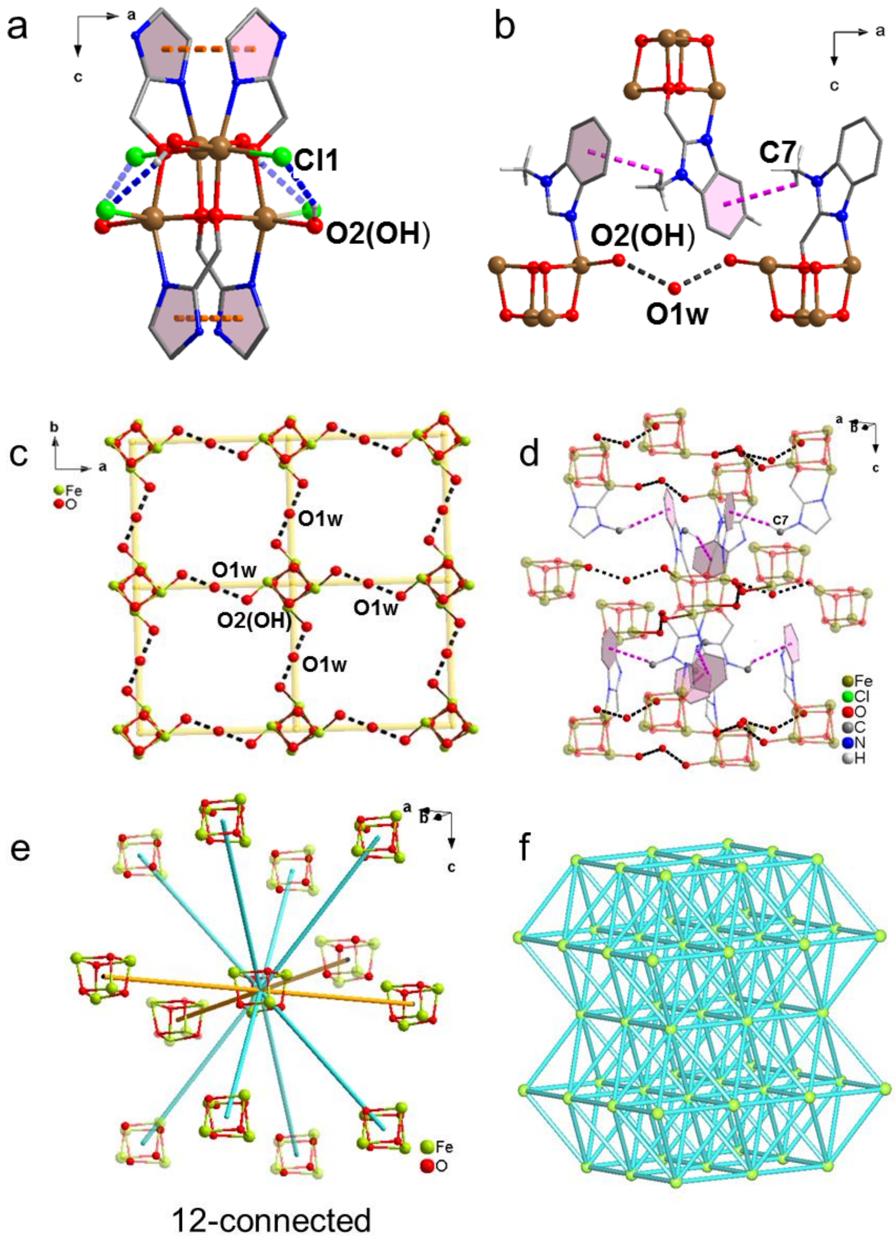

Figure S3. Supramolecular interactions within the crystal structure of **1-2d**. (a) O(MeOH)–H···Cl H-bond (blue) and π···π intracluster (light orange); (b) C–H···π (pink) interactions and O–H···O hydrogen bond (black dotted line); (c) O–H···O hydrogen bond (black dotted line) connected intercluster (4, 4) net in the structure of **1-2d.** (d) C(Me)–H···π intercluster interaction (pink dotted line) and O–H···O hydrogen bond (black dotted line) in the structure of **1-2d**; (e) 8-connected intercluster through C(Me)–H···π interaction (aqua solid line, 12.02 Å) and O–H···O hydrogen bond (light orange solid line, 10.96 Å). (f) Uninodal 12-connected **fcu** net of **1-2d.**

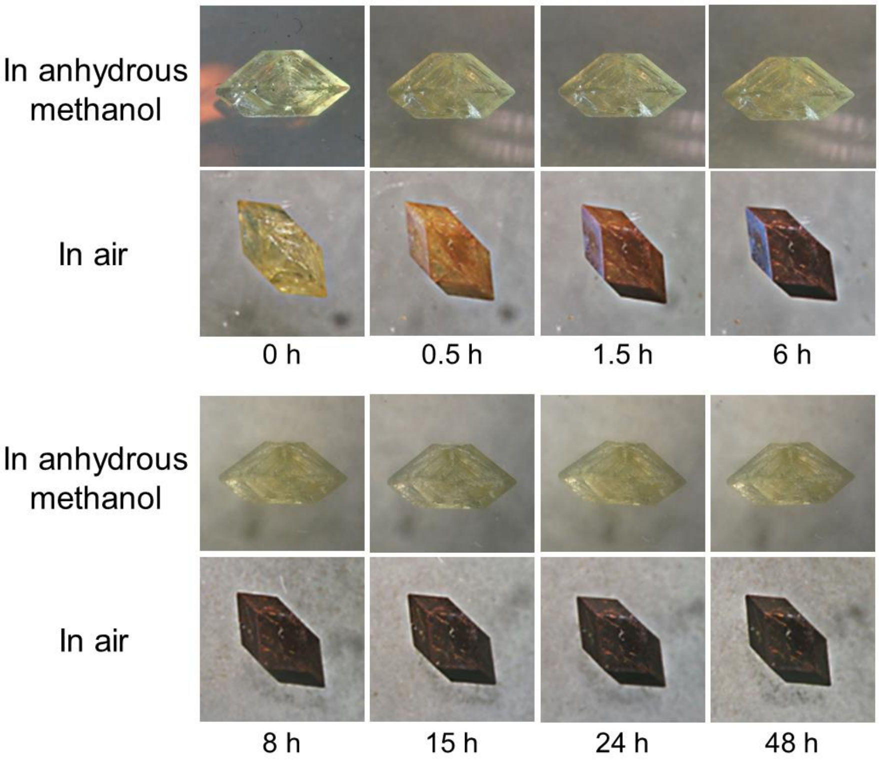

Figure S4. Time-dependent optical photography of two crystals of **1**; one immersed in anhydrous methanol (top) and the other exposed to air under ambient condition (bottom).

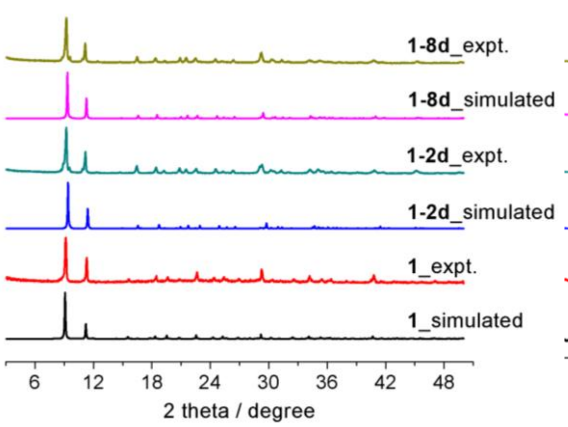

(a)

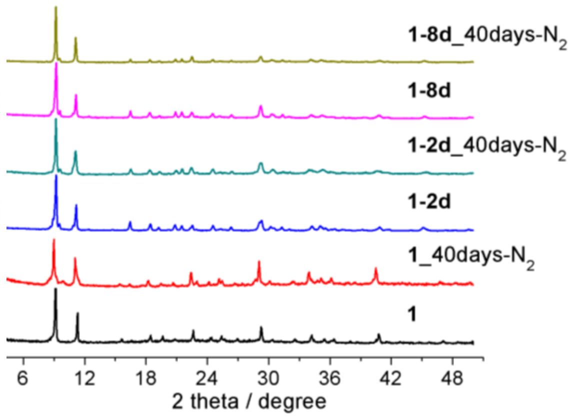

(b)

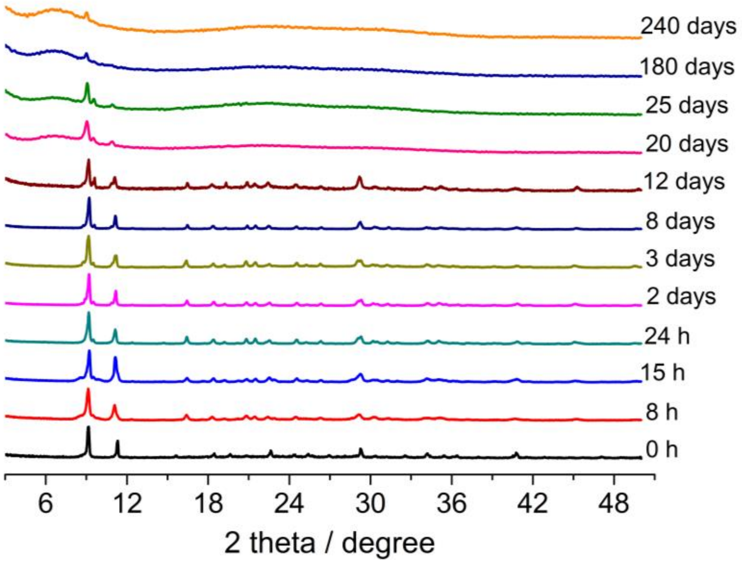

(c)

Figure S5. (a) Experimental and simulated PXRD patterns of **1**, **1-2d** and **1-8** (left); (b) those kept under N_2 to exclude O_2 and water for 40 days (right); (c) Time-dependent PXRD patterns of **1**.

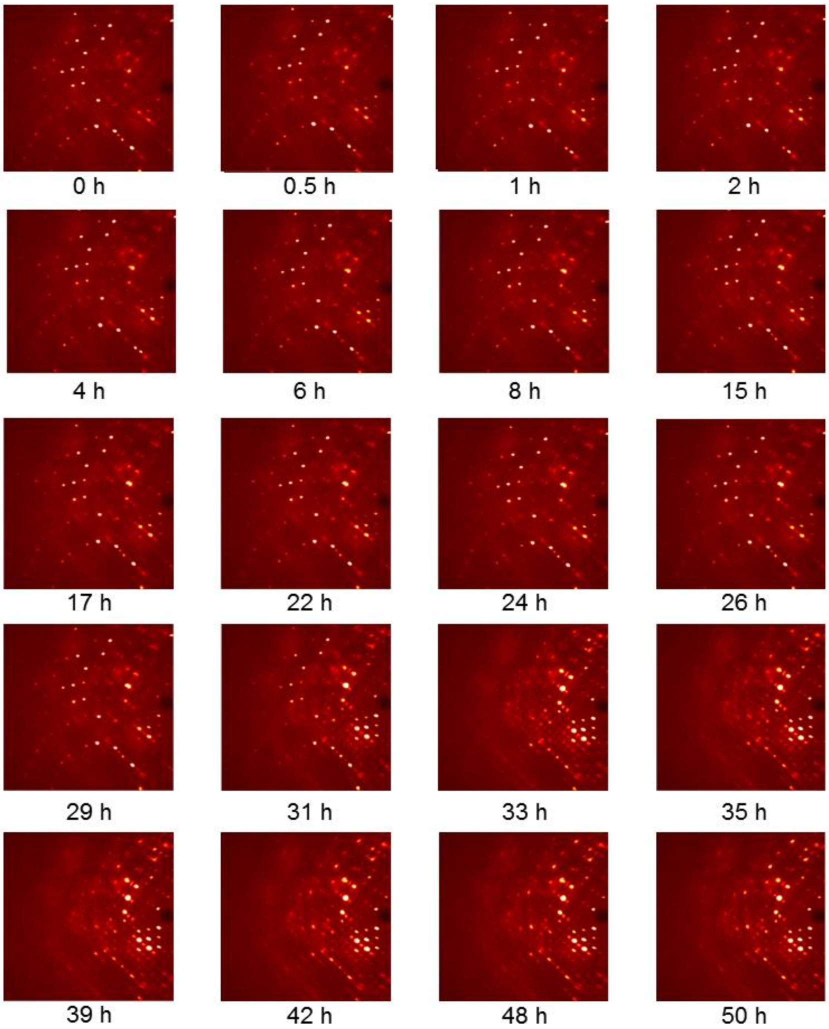

Figure S6. Diffraction frames for a single crystal of **1** as a function of exposure time in air.

Table S4. SC-SC transformation involves from discrete cluster to discrete cluster with chemical changes on the molecular.

C1: Change of oxidation state of metal
C2: Swapping original coordinated ion on metal coordination sphere.
C3: Change of coordinated ligand
C4: Change of uncoordinated guest or counter ion

SC-SC	C1	C2	C3	C4	Ref.
**Co-based SC-SC transformation (3)**					
$NHEt_3[Co^{II}_6$(phendc[a])$_6$ Cl] ·MeOH 3H_2O→H[Co^{II}_6(phendc)$_6$ Cl]					
	No	No	No	**Yes**	1a
2{[Co^{II}(hep-H[b])$_2(H_2O)_4$] ·SO_4}→[Co^{II}_2(hep-H)$_2(H_2O)_4(\mu_2$-$SO_4)_2$]					
	No	No	**Yes**	**Yes**	1b
[{(bpbp[c])$Co^{II}_2(NO_3)$}$_2$(NH_2bdc[d])]$(NO_3)_2$·2H_2O→[{(bpbp)$Co^{III}_2(O_2)$}$_2$(NH_2bdc)]$(NO_3)_4$·7H_2O					
	**Yes**	No	**Yes**	**Yes**	1c
**Cu-based SC-SC transformation (4)**					
[$Cu^{II}_3(L_1$[e]$)_2(EtNH_2)_2(MeOH)_2$] ·2CH_3OH $CHCl_3$→2[$Cu^{II}(HL_1)(EtNH_2)$]					
	No	No	**Yes**	**Yes**	2a
2[$Cu^{II}(\mu_2$-hep)(TFA[f])$(H_2O)]_2$→[$Cu^{II}_4(\mu_3$-hep)$(\mu_2$-hep)$(\mu_2$-TFA$)_2$(TFA)$_2$]					
	No	No	**Yes**	**Yes**	2b
2[$Cu^{II}(NH_3)_3(L_6$[g])] ·0.66H_2O→[$Cu^{II}_2(NH_3)_6(L_6$[f]$)_2$]					
	No	No	**Yes**	**Yes**	2c
[Cu^{II}(OAc) (hep)$_2$Cu(OAc)] ·2H_2O→[Cu^{II}(OAc) (hep)$_2$Cu(OAc)]					
	No	No	No	**Yes**	2d
**Zn-based SC-SC transformation (1)**					
2[$ZnBr_2$(4spy)[h]$_2$]→[Zn_2Br_4(*rctt*-ppcb)[i](4spy)$_2$]					
	No	No	No	No	3
**Mo-based SC-SC transformation (1)**					
[(DAniF)[j]$_3Mo^{III}_2(\mu$-OH)$_2Mo^{III}_2$(DAniF)$_3$]→[(DAniF)$_3Mo^{IV}_2(\mu$-O)$_2Mo^{IV}_2$(DAniF)$_3$]					
	**Yes**	No	No	No	4
**Rh-based SC-SC transformation (3)**					
[Rh(N_2)(SIPr)[k]$_2$Cl]→[Rh(O_2)(SIPr)$_2$Cl] (gas exchange)					
	No	No	**Yes**	No	5
[Rh(O_2)(SIPr)$_2$Cl]→[Rh(CO)(SIPr)$_2$Cl] (gas exchange)					
	No	No	**Yes**	No	5
[Rh(N_2)(SIPr)$_2$Cl]→[HRh(SIPr-H)(SIPr)Cl]					
	No	No	**Yes**	No	5
**Ag-based SC-SC transformation (1)**					
{$Ag_2(PPh_3)$[l]$_6$[*trans*-$B_{20}H_{18}$]}→{$Ag_2(PPh_3)_6$[*iso*-$B_{20}H_{18}$]}					
	No	No	No	No	6
**Au-based SC-SC transformation (1)**					
[$Au_{10}(\mu_3$-S$)_4(\mu$-vdpp)[m]$_4$] ·$(PF_6)_2$ ·Et_2O ·$(Et_3N)_3$→[$Au_{12}(\mu_3$-S$)_4(\mu$-vdpp$)_2(\mu$-dppes)[n]$_2$] Cl					

	No	No	**Yes**	**Yes**	7
**Ce-based SC-SC transformation (1)**					
$[Ce^{III}(Homtaa)^{o}(omtaa)] \rightarrow [Ce^{IV}(omtaa)_2]$	**Yes**	No	No	No	8
**Dy-based SC-SC transformation (1)**					
$\{[Dy^{III}(L_1)^{p}{}_2(acac)^{q}{}_2]NO_3\} \cdot CH_3OH \cdot 3H_2O \rightarrow \{[Dy^{III}(L_1)_2(acac)_2]NO_3\} \cdot CH_3OH \cdot 3H_2O$	No	No	No	**Yes**	9
**Mixed-Metal-based SC-SC transformation (8)**					
$[Ag_{30}({}^{t}BuS)^{r}{}_{20}V^{V}{}_{10}V^{IV}{}_2O_{34}] \cdot 10CH_3OH \rightarrow [Ag_{30}({}^{t}BuS)_{20}V^{V}{}_{10}V^{IV}{}_2O_{34}] \cdot 7CH_3OH$	No	No	No	**Yes**	10a
$K_2[Cr^{III}{}_3O(OOCH)_6(mepy)^{s}{}_3]_2[\alpha\text{-}SiW_{12}O_{40}] \cdot 8H_2O \rightarrow$ $(mepyH)_2[Cr^{III}{}_3O(OOCH)_6(mepy)_2(H_2O)]_2[\alpha\text{-}SiW_{12}O_{40}] \cdot 8H_2O$	No	No	No	**Yes**	10b
$\{[Fe^{II}(Tp)^{t}(CN)_3]_2[Co(4,4'\text{-}bcbpy)^{u}{}_2]_2\}(ClO_4)_2 \cdot 2CH_3OH \rightarrow$ $\{[Fe^{II}(Tp)(CN)_3]_2[Co(4,4'\text{-}bcbpy)_2]_2\}$	No	No	No	**Yes**	10c
$\{({}^{HS}Fe^{II})_3({}^{HS}Fe^{III})_6(W^{IV})_6(CN)_{48}](MeOH)_{24}\} \cdot 12.5MeOH$ $\rightarrow \{({}^{HS}Fe^{III})_6({}^{HS}Fe^{II})_3(W^{IV})_6(CN)_{48}](MeOH)_{24}\} \cdot 18MeOH$	**Yes**	No	No	**Yes**	10d
$[Co^{II}{}_2Dy^{III}(L^{Br})^{v}{}_2(H_2O)] \cdot NO_3 \cdot 3H_2O \rightarrow [Co^{II}{}_2Dy^{III}(L^{Br})_2(H_2O)] \cdot NO_3$	No	No	No	**Yes**	10e
$[CuL^{w}Gd(NO_3)_3] \rightarrow [CuLGd(\mu_2\text{-}NO_3)(NO_3)_2]$	No	No	**Yes**	No	10f
$[CuLTb(NO_3)_3] \rightarrow [CuLTb(\mu_2\text{-}NO_3)(NO_3)_2]$	No	No	**Yes**	No	10f
$[CuLTb(NO_3)_3] \rightarrow [CuLTb(\mu_2\text{-}NO_3)(NO_3)_2]$	No	No	**Yes**	No	10f
**Fe-based SC-SC transformation (5)**					
$[Fe^{III}{}_3(\mu_3\text{-}O)(\mu_2\text{-}CH_3COO)_6(C_5H_5NO)_2(H_2O)]ClO_4 \cdot 3H_2O \rightarrow$ $Fe^{III}{}_3(\mu_3\text{-}O)(\mu_2\text{-}CH_3COO)_6(C_5H_5NO)_2(CH_3OH)]ClO_4 \cdot 3H_2O$	No	No	No	**Yes**	11a
$[Fe^{II}(tpa)^{x}\{N(CN)_2\}]_4 \cdot (BF_4)_4 \cdot 2H_2O \rightarrow [Fe^{II}(tpa)\{N(CN)_2\}]_4 \cdot (BF_4)_4$	No	No	No	**Yes**	11b
$[Fe^{II}{}_2(\mu_2\text{-}L)^{y}{}_3(L)_2(NCS)_4] \cdot 2CH_3CH_2OH \rightarrow [Fe^{II}{}_2(\mu_2\text{-}L)_3(L)_2(NCS)_4] \cdot 2CH_3CH_2OH \cdot 1.5H_2O$	No	No	No	**Yes**	11c
$[Fe^{III}{}_3(\mu_3\text{-}O)(\mu_2\text{-}CH_3COO)_6(C_5H_5NO)_2(H_2O)]ClO_4 \cdot 3H_2O \rightarrow$ $[Fe^{III}{}_3(\mu_3\text{-}O)(\mu_2\text{-}CH_3COO)_6(C_5H_5NO)_2(C_5H_5N)]ClO_4 \cdot C_5H_5N$	No	No	**Yes**	**Yes**	11d
$[Fe^{III}(qsal)^{z}(thsa)^{aa}] \cdot 0.5CH_3CN \rightarrow [Fe^{III}(qsal)(thsa)]$	No	No	No	**Yes**	11e

$[Fe^{II}_4(mbm)_4(Cl)_4(CH_3OH)_4] \rightarrow [Fe^{III}_4(mbm)_4(OH)_4(Cl)_4]\cdot 2H_2O$					
	**Yes**	**Yes**	**Yes**	**Yes**	**This Work**:

[a]H_2phendc = 1,10-phenanthrolin-2,9-dicarbaldehyde-2,9-dicarboxylate. [b]hep-H =2-(2-hydroxyethyl) pyridine. [c]bpbp$^-$ = 2,6-bis(N,N-bis(2-pyridylmethyl)-aminomethyl)-4-tert-butylphenolato [d]NH_2bdc = 2-amino-1,4-benzenedicarboxylato). [e]L_1 = N-(3-tert-butyl-2-hydroxybenzylidene)- 4,6-O-ethylidene-β-D-glucopyranosylamine). [f]TFA-H = trifluoroacetic acid. [g]H_2L^6 = 2,2'-(1,2-phenylene bis(methylene))bis(sulfanediyl)-dibenzoic acid). [h]4spy = *trans*-4-styrylpyridine. [i]*rctt*-ppcb = *rctt*-1,3- bis(4'-pyridyl)-2,4-bis(phenyl)cyclobutane. [j]DAniF = (*p*-anisyl)NC(H)N(*p*-anisyl). [k]SIPr = *N,N'*- (2,6-$iPr_2C_6H_3)_2C_3H_4N_2$. [l]PPh = triphenylphosphine. [m]vdpp = Vinylidenebis(diphenylphosphine). [n] dppes = bis(2,2-bis(diphenylphosphino)ethyl)sulfane. [o]H_2omtaa = octamethyltetraazaannulene. [p]L_1 = (-)-4,5-bis (pinene)-2,2'-bipyridine. [q]acac = acetylacetonate. [r] *t*BuS = *tert*-butyl mercaptan. [s] mepy = 4-methylpyridine. [t]Tp = hydrotris(pyrazolyl)borate. [u]4,4'-bcbpy = 4,4'-bis(ethoxycarbonyl)-2,2'-bipyridine. [v]L^{Br} = 2,2,2 ″ -(((nitrilotris(ethane-2,1-diyl))tris(azanediyl))tris(methylene))tris-(4-bromophenol)). [w]H_2L = (*R,R*)-*N,N'*-bis-(3-methoxysalicylidene)cyclohexane-1,2-diamine. [x]tpa = tris(2-pyridylmethyl)amine. [y]L = 4-benzene-1,2,4-triazole. [z]qsal = quinolylsalicylaldimine. [aa]thsa = thiosemicarbazone-salicylaldimine.

(2) References

(3) (1) (a) Chen, Q.; Zeng, M.-H.; Wei, L.-Q.; Kurmoo, M. *Chem. Mater.* **2010**, *22*, 4328-4334. (b) Mobin, S. M.; Mohammad, A. *Dalton Trans.*, **2014**, *43*, 13032-13040. (c) Bhattacharya, S.; Natarajan, S. *Z. Anorg. Allg. Chem*. **2014**, *640*, (14), 2922-2930. (c) Sundberg, J.; Cameron, L. J.; Southon, P. D.; Kepert, C. J.; Mckenzie, C. J. *Chem. Sci.*, **2014**, *5*, 4017-4025.

(4) (2) (a) Sah, A. K.; Tanase, T. *Chem. Commun*., **2005**, *48*, 5980-5981. (b) Mobin, S. M.; Srivastava, A. K.; Mathur, P.; Lahiri, G. K. *Inorg. Chem.* **2009,** *48*, 4652-4654. (c) Sun, J.; Dai, F.; Yuan, W.; Bi, W.; Zhao, X.; Sun, W.; Sun, D. *Angew. Chem. Int. Ed*. **2011**, *50*, 7061-7064. (d) Mobin, S. M.; Srivastava, A. K.; Mathur, P.; Lahiri, G. K. *Dalton. Tran.* **2010,** *39*, 1447-1449.

(5) (3) Medishetty, R.; Yap, T. T. S.; Koh, L. L.; Vittal, J. J. *Chem. Commun*., **2013**, *49*, 9567-9569.

(6) (4) Cotton, F. A.; Li, Z.; Murillo, C. A.; Wang, X.; Yu, R.; Zhao, Q.; *Inorg. Chem*. **2007**, *46*, 3245-3250.

(7) (5) Zenkina, O. V.; Keske, E. C.; Wang, R.; Crudden, C. M. *Angew. Chem. Int. Ed*. **2011**, *50*, 8100-8104.

(8) (6) Avdeeva, V. V.; Buzin, M. I.; Malinina, E. A.; Kuznetsova, N. T.; Vologzhanina, A. V. *CrystEngComm*, **2015**, *17*, 8870-8875.

(9) (7) Yao, L.-Y.; Hau, F. K.-W.; Yam, V. W.-W. *J. Am. Chem. Soc*. **2014**, *136*, 10801-10806.

(10)(8) Williams, U. J.; Mahoney, B. D.; Lewis, A. J.; DeGregorio, P. T.; Carroll, P. J.; Schelter, E. J. *Inorg. Chem*. **2013**, *52*, 4142-4144.

(11)(9) Liu, J.; Zhang, X.-P.; Wu, T. Ma, B.-B.; Wang, T.-W.; Li, C.-H.; Li, Y.-Z.; You, X.-Z. *Inorg. Chem*. **2012**, *51*, 8649-8651.

(12)(10) (a) Liu, H.; Song, C.-Y.; Huang, R.-W.; Zhang, Y.; Xu, H.; Li, M.-J.; Zang, S.-Q.; Gao, G.-G. *Angew. Chem. Int. Ed.* **2016**, *55*, 3699-3703. (b) Uchida, S.; Mizuno, K.; Kawahara, R.; Takahashi, E.; Mizuno, N. *Chem. Lett*. **2014**, *43*, 1192-1194. (c) Cao, L.; Tao, J.; Gao, Q.; Liu, T.; Xia, Z.; Li, D. *Chem. Commun*., **2014**, *50*, 1665-1667. (d) Chorazy, S.; Podgajny, R.; Nogaś, W.; Nitek, W.; Kozieł, M.; Rams, M.; Juszyńska-Gałązka, E.; Żukrowski, J.; Kapusta, C.; Nakabayashi, K.; Fujimoto, T.;

Ohkoshi, S.; Sieklucka, B. *Chem. Commun*., **2014**, *50*, 3484-3487. (e) Liu, J.-L.; Wu, J.-Y.; Huang, G.-Z.; Chen, Y.-C.; Jia, J.-H.; Ungur, L.; Chibotaru, L. F.; Chen, X.;-M.; Tong, M.-L. Scientific Reports, 2015, 5, 16621-16629. (f) Wen, H.-R.; Bao, J.; Liu, S.-J.; Liu, C.-M.; Zhang, C.-W.; Tang, Y.-Z. *Dalton Trans.*, **2015**, *44*, 11191-11201.

(13)(11) (a) Supriya, S.; Das, S. K. *J. Am. Chem. Soc*. **2007**, *129*, 3464-3465. (b) Wei, R.-J.; Huo, Q.; Tao, J.; Huang, R.-B.; Zheng, L.-S. *Angew. Chem. Int. Ed.* **2011**, *50*, 8940-8943. (c) Wu. X. X.; Wang, Y. Y.; Yang, P.; Xu, Y. Y.; Huo, J. Z; Ding, B.; Wang, Y.; Wang, X. *Cryst. Growth Des.* **2014**, *14*, 477-490. (d) Supriya, S.; Das, S. K. *CrystEngComm* **2015**, *17*, 8850-8857. (e) Phonsri, W.; Davies, C. G.; Jameson, G. N. L.; Moubaraki, B.; Murray, K. *Chem. Eur. J.* **2016**, *22*, 1322-1333.

Magnetic Properties

The change of oxidation state of the Fe centres from Fe(II) to Fe(III) is accompanied by a change of the spin state from S = 2 to 5/2 and the orbital part from L = 2 to 0. Thus, major changes to the magnetic properties are expected. The magnetic susceptibilities were measured in 1 kOe (Figures 4, S10). $\chi_m T$ for **1** at 300 K of 14.0 cm^3 K mol^{-1} is higher than the spin-only value of 12.0 cm^3 K mol^{-1} expected for four magnetically isolated high-spin Fe(II) ions (S = 2, g = 2), indicating the importance of orbital contribution for Fe(II). As the temperature is lowered, $\chi_m T$ gradually increases to 15.7 cm^3 K mol^{-1}, followed by a sharp decrease below *ca*. 15 K reaching 8.8 cm^3 K mol^{-1} at 2 K. Between 30 and 300 K χ_m obeys the Curie–Weiss law with the θ value of +2.8(2) K, indicating the presence of overall near-neighbour ferromagnetic coupling.

The susceptibility of **1** was fitted using the PHI program with a model having two independent exchange interactions corresponding to the S_4 symmetry observed in the structure (Figure S7a). The best fit assuming Fe(II) with S = 2, gives g = 2.14(1), D = 14.3(1) cm^{-1}, E = 2.1(1) cm^{-1}, and two of the six exchange interactions are antiferromagnetic with J_1 = −0.63(1) cm^{-1}, while the remaining four interactions are ferromagnetic with J_2 = 0.63(1) cm^{-1}, confirming the overall ferromagnetic behaviour as indicated by the small positive θ value of +2.8(2) K. DFT calculations using the single-crystal data without optimization estimate J_1/cm^{-1} = –4.2 for **1** and –9.1 for **1-2d**, and J_2/cm^{-1} = 7.4 for **1** and –4.5 for **1-2d**. (Table S6). The fitting results for **1** are comparable to those reported for cubic complexes having the $\{Fe^{II}_4\}$ core, supporting the divalent nature of iron in **1**. In contrast, $\chi_m T$ for **1-2d** decreases gradually from 9.3 cm^3 K mol^{-1} at 300 K to 1.8 cm^3 K mol^{-1} at 2 K, and the Curie-Weiss fitting of $1/\chi_m$ gives θ = –53.0(2) K, implying the overall antiferromagnetic exchange which will be favoured by Fe(III) ions in **1-2d**.

To track the evolution of the magnetic properties we prepared a large batch of crystals from which small quantities were taken at different exposure times up to 180 days for magnetization measurements (Figures 4). Because the structures (hence the molar weights) are different for each state, we switch from molar susceptibility to gram susceptibility for better comparison. $\chi_g T$ progressively decreases with exposure time. After 8 h $\chi_g T$ becomes almost flat with temperature implying the weakening of the ferromagnetic interactions (J_2). By 15 h, $\chi_g T$ continuously decreases with lowering temperature, indicating the overall near-neighbor exchange is now antiferromagnetic (θ = −25.9(2) K). Beyond 15 h, $\chi_g T$ curves get lower for longer exposure times, implying the strength of the antiferromagnetic coupling increases gradually up to 180 days. While the high temperature data give information on the dominant coupling, the sharp decrease of $\chi_g T$ value below 30 K for **1** relates to the zero field splitting (ZFS) of Fe(II) ions. Thus, the sharp decrease for 8 h $\chi_g T$ data indicates that Fe(II) ions still present in the sample but it is proportionally less for 15 h sample. The isothermal magnetization at 2 K (Figure 4) exhibits an increase to saturation for the virgin sample **1**, but for those exposed to air the saturation is not reached. This is consistent with the temperature dependence data discussed above where progressive increase of the AF coupling is observed as a function of exposure time.

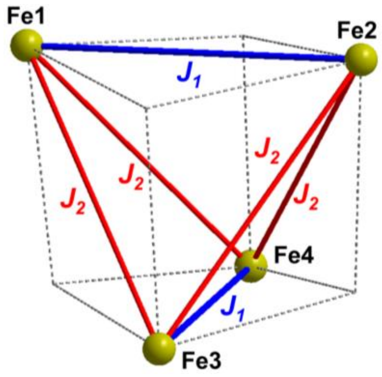

(a)

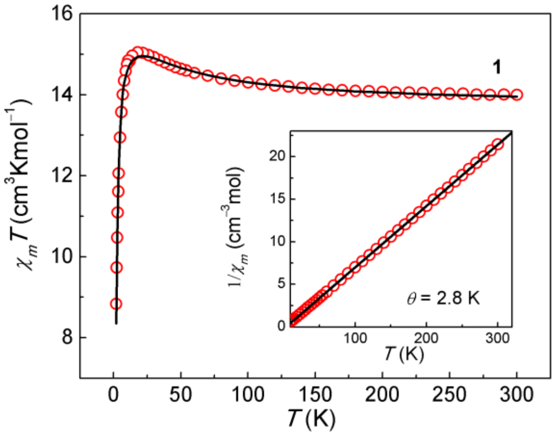

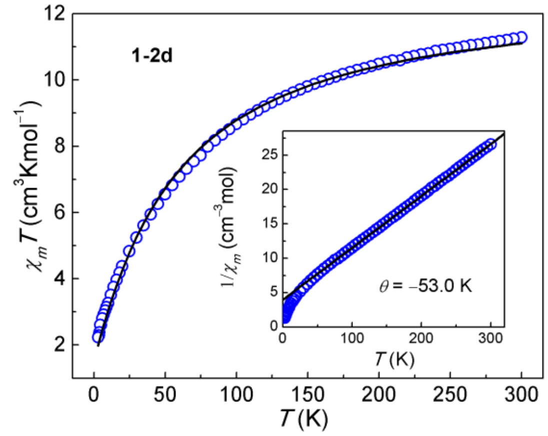

(b) (c)

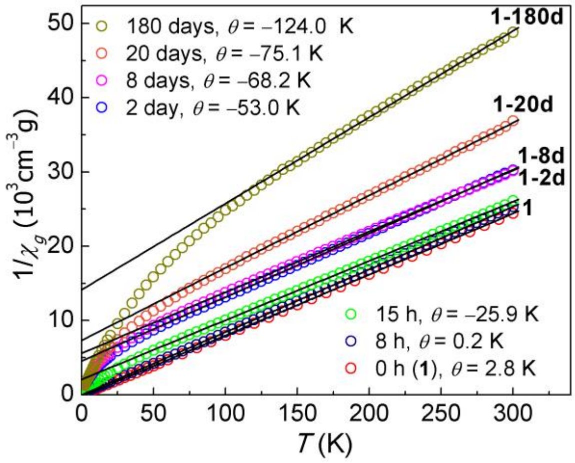

(d)

Figure S7. (a) The model used to simulate the magnetic susceptibility data; (b) Temperature dependence of $\chi_m T$ and $1/\chi_m$ (inset) for **1** (H = 1 kOe) in the unit of molar susceptibility; (c) Temperature dependence of χ_mT and $1/\chi_m$ (inset) for **1-2d** (H = 1 kOe) in unit of molar susceptibility; (d) $1/\chi_m$ *vs* T for **1** after exposure to air for different times. Solid lines are the Curie-Weiss fits.

Bond lengths and bond angles determine the nature and strength of exchange interactions. Considering the bond lengths and bond angles for **1**, **1-2d** and **1-8d**, the interactions between iron ions can be divided into two groups, as illustrated in Figure S7a. A general spin Hamiltonian can be imployed to describe the magnetic hehaviors, preasumming all the Fe ions have identical magnetic properties (*Chem. Commun.*, **2012**, *48*, 2430–2432).

$$\hat{H} = -2J_1(\hat{\mathbf{S}}_1 \cdot \hat{\mathbf{S}}_2 + \hat{\mathbf{S}}_3 \cdot \hat{\mathbf{S}}_4) - 2J_2(\hat{\mathbf{S}}_1 \cdot \hat{\mathbf{S}}_3 + \hat{\mathbf{S}}_1 \cdot \hat{\mathbf{S}}_4 + \hat{\mathbf{S}}_2 \cdot \hat{\mathbf{S}}_3 + \hat{\mathbf{S}}_2 \cdot \hat{\mathbf{S}}_4)$$

$$+\sum_{i=1}^{4} g\mu_B \mathbf{B} \cdot \hat{\mathbf{S}}_i + D(\hat{S}_{i,z}^2 - \hat{\mathbf{S}}_i^2/3) + E(\hat{S}_{i,x}^2 - \hat{S}_{,}^2 \qquad \text{(S1)}$$

where the symbols have their standard meaning and electron spin S = 2 for Fe(II) ions.

Table S5. Results of fitting of the $\chi_g T$ *versus T* curves.

Samples	Oxidation state	J_1/J_2 (cm^{-1})	C (cm^3 K mol^{-1})	θ (K)[a]
**1**	Fe(II)	−0.63(1)/ 0.63(1)	13.87(2)	2.8(2)
**1-8h**	Fe(II/III)	N.D. [b]	13.48(2)	0.2(2)
**1-15h**	Fe(II/III)	N.D. [b]	14.28(2)	−25.9(2)
**1-2d**	Fe(III)	−1.92(5)/ −1.92 (5)	13.26(2)	−53.0(2)
**1-8d**	Fe(III)	−2.30(5)/ −2.30 (5)	13.89(2)	−68.2(2)
**1-20d**	Fe(III)	−3.24(5)/ −3.24 (5)	11.67(2)	−75.1(2)
**1-180d**	Fe(III)	−7.97(5)/ −7.97 (5)	9.80(2)	−124.0(2)

[a] Fitting details are shown in Figure 4a. [b] Not determined.

Computational Methodology.

The spin state and the nature of the magnetic exchange interaction (J), different electron configuration of Fe based on the single-crystal atomic coordinates of **1** without optimization were calculated with B3LYP functional adding D3 version of Grimme's dispersion with Becke-Johnson damping and 6-311G(d) basis set for light elements and SDD or LANL2DZ ECP for Fe. J for **1** and **1-2d** were calculated using Noodleman's broken symmetry model.[1-4] The geometries of **1** and **1-2d** clusters as well as their derivatives with Cl or CH_3OH/OH or ligand removed were full optimized with the same functional and basis sets. Gibbs free energy surfaces for **1** and **1-2d** were then built. Complexation energies between clusters were calculated with counterpoise method with basis set superposition error (BSSE) being corrected. All calculations were performed using Gaussian 09 software.[5] DFT calculations using the single-crystal data without optimization estimate J_1 /cm^{-1} = –4.2 for **1** and –9.1 for **1-2d**, and J_2/cm^{-1} = 7.4 for **1** and –4.5 for **1-2d**.

(14)(1) Noodleman, L. *J. Chem. Phys.* **1981**, *74*, 5737-5743.

(15)(2) Noodleman, L.; Davidson, E. R. Chem. Phys. **1986**, *109*, 131-143.

(16)(3) Noodleman, L.; Case, D. A.; Aizman, A. J. *J. Am. Chem. Soc.* **1988**, *110*, 1001-1005.

(17)(4) Noodleman, L.; Case, D. A. Adv. Inorg. Chem. **1992**, *38*, 423-470.

(18)(5) (Gaussian 09, Revision D.01, Frisch, M. J.; Trucks, G. W.; Schlegel, H. B.; Scuseria, G. E.; Robb, M. A.; Cheeseman, J. R.; Scalmani, G.; Barone, V.; Mennucci, B.; Petersson, G. A.; Nakatsuji, H.; Caricato, M.; Li, X.; Hratchian, H. P.; Izmaylov, A. F.; Bloino, J.; Zheng, G.; Sonnenberg, J. L.; Hada, M.; Ehara, M.; Toyota, K.; Fukuda, R.; Hasegawa, J.; Ishida, M.; Nakajima, T.; Honda, Y.; Kitao, O.; Nakai, H.; Vreven, T.; Montgomery, J. A., Jr.; Peralta, J. E.; Ogliaro, F.; Bearpark, M.; Heyd, J. J.; Brothers, E.; Kudin, K. N.; Staroverov, V. N.; Keith, T.; Kobayashi, R.; Normand, J.; Raghavachari, K.; Rendell, A.; Burant, J. C.; Iyengar, S. S.; Tomasi, J.; Cossi, M.; Rega, N.; Millam, J. M.; Klene, M.; Knox, J. E.; Cross, J. B.; Bakken, V.; Adamo, C.; Jaramillo, J.; Gomperts, R.; Stratmann, R. E.; Yazyev, O.; Austin, A. J.; Cammi, R.; Pomelli, C.; Ochterski, J. W.; Martin, R. L.; Morokuma, K.; Zakrzewski, V. G.; Voth, G. A.; Salvador, P.; Dannenberg, J. J.; Dapprich, S.; Daniels, A. D.; Farkas, O.; Foresman, J. B.; Ortiz, J. V.; Cioslowski, J.; and Fox, D. J. Gaussian, Inc., Wallingford CT, **2013**.

Table S6. Energies of electronic states for **1** and **1-2d** based on experimental geometry without optimization.

Electronic States	Number of unpaired electron on each Fe	Relative Energy (cm^{-1})
**1-** Low Spin	0	47379.7
**1** Middle Spin	2 2 2 2	34210.8
**1** High Spin	4 4 4 4	0.0
**1**-Broken Symmetry1	4 4 -4 -4	476.5
**1** -Broken Symmetry2	4 -4 4 -4	103.4
**1** -Broken Symmetry3	4 4 4 -4	-1499.0
**1-2d** High Spin	5 5 5 5	0
**1-2d** -Broken Symmetry1	5 5 -5 -5	-288.2
**1-2d** -Broken Symmetry2	5 -5 5 -5	-435.4
**1-2d** -Broken Symmetry3	5 5 5 -5	-289.5

HF-EPR measurements

High frequency/field electron paramagnetic resonance (HF-EPR) were performed on locally developed instruments in pulsed-magnetic fields.[1] The samples of **1**, **1-2d**, **1-8d** and **1-180d** were gently crushed and pressed hard to avoid the movements of sample due to the strong field torque during the EPR measurements.

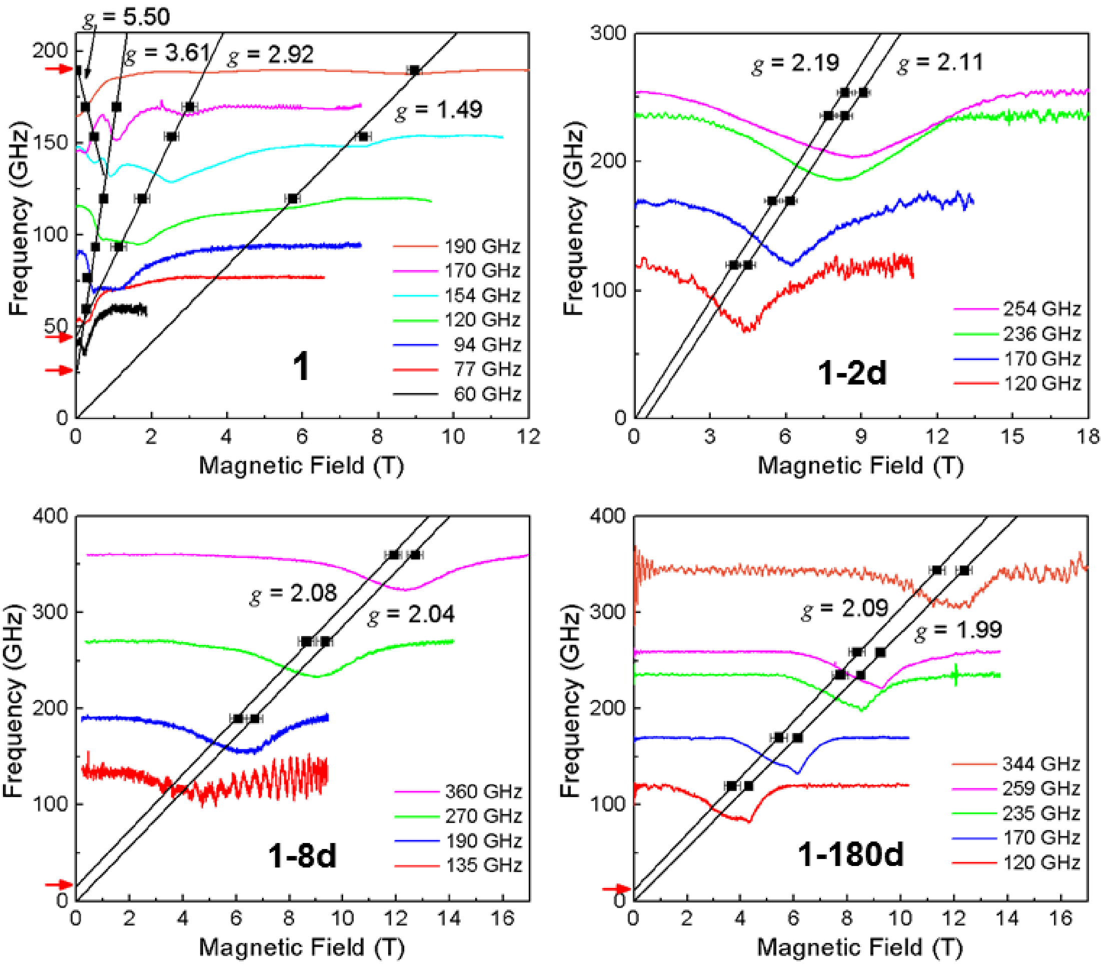

Figure S8. HF-EPR spectra of **1**, **1-2d**, **1-8d**, and **1-180d** at various frequencies. The spectra are offset in proportion to the frequency. Black dots represent the resonance fields for each spectrum. Solid lines are the linear fit to each resonance branch. Red arrows indicate the zero-field splitting (gaps).

(19) (a) Nojiri, H.; Choi, K.-Y.; Kitamura, N.; *J. Magn. Magn. Mater.* 2007, *310*, 1468-1472. (b) Nojiri, H.; Ouyang; Z.-W. *Thz. Sci. Techn.* 2012, *5*, 1-10.

^{57}Fe Mössbauer spectroscopy

Zero-field ^{57}Fe Mössbauer spectroscopy has been used to establish the oxidation and spin states of the local Fe sites of the clusters contained in the **1**, **1-8h**, **1-15h**, **1-2d** and **1-180d** samples. Thus, these measurements allowed us to monitor the relative ratio of ferric to ferrous ions. The spectra recorded at 80 K reveals major changes (Figure 4c and Table S7). Our best single-site simulation of the 80 K, 0 T spectrum of **1-2d** is shown in Figure S9a. In order to resolve the discrepancy between the experimental and simulated spectra we have included in our simulation a distribution of ΔE_Q values (Figure S9b, Table S8). Thus, these simulations **1-2d** and **1-180d** demonstrate the presence of two distinct high-spin iron(III) sites that are characterized by nearly identical isomer shifts and distinctly different quadrupole splitting values (Figure S9 and Table S9).

As shown in figure 4c, for the initial state (**1**) we observe a well-defined quadrupole doublet characterized by an isomer shift δ = 1.22(1) mm/s, quadrupole splitting ΔE_Q = 3.25(1) mm/s (Table S16, site 1). These parameters are typical of high-spin ferrous ions that have an oxygen-rich octahedral coordination environment.[42] After 2 days, the oxidation of ferrous to ferric ions is essentially complete. The spectra for the all-ferric samples, **1-2d** to **1-180d** could be satisfactorily simulated by considering only two distinct sites with nearly identical isomer shifts but distinct quadrupole splitting values (Table S9, site 4 and 5). Knowledge of the initial and final species offers us the opportunity to assess putative intermediates that might be present in samples with an intermediate air-exposure time. The spectrum of **1-8h** has been simulated using five sites (Table S9). Site 1 and site 2, with δ = 1.22(1) mm/s, ΔE_Q = 3.25(1) mm/s and δ = 1.22(1) mm/s, ΔE_Q = 2.94(1) mm/s, consistent with the parameters used for the ferrous compound.[1,2] The isomer shift and quadrupole splitting of the other 3 sites (Table S9, site 3-5) are in accordance with of the parameters used for the ferric compounds. Prolonged exposure to air leads to a progressive increase in the fraction of high-spin ferric ions such that after 15 h they account for 34% of the sample.

(1) Arroyave, A., Lennartson, A., Dragulescu-Andrasi, A., Pedersen, K. S., Piligkos, S., Stoian, S. A., Greer, S. M., Pak,C., Hietsoi, O., Phan, H., Hill, S., McKenzie, C. J. & Shatruk, M. Spin crossover in Fe(II) complexes with N_4S_2 coordination. *Inorg. Chem.* **55**, 5904−5913 (2016).

(2) Boudalis, A. K., Clemente-Juan, J.-M., Dahan, F. & Tuchagues, J.-P. New poly-iron(II) complexes of N_4O dinucleating Schiff bases and pseudohalides: Syntheses, structures, and magnetic and Mössbauer properties. *Inorg. Chem.* **43**, 1574–1586 (2004).

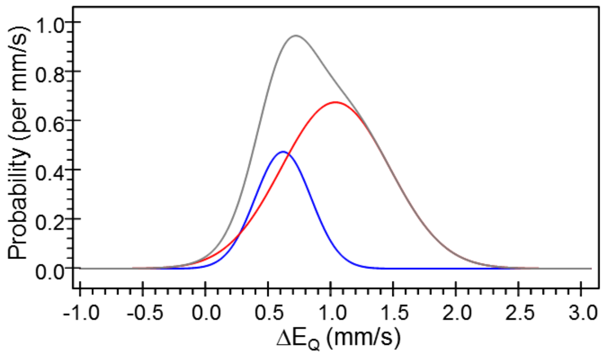

(b)

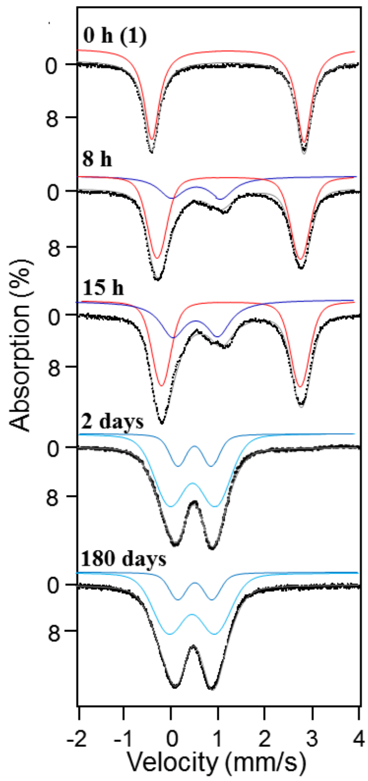

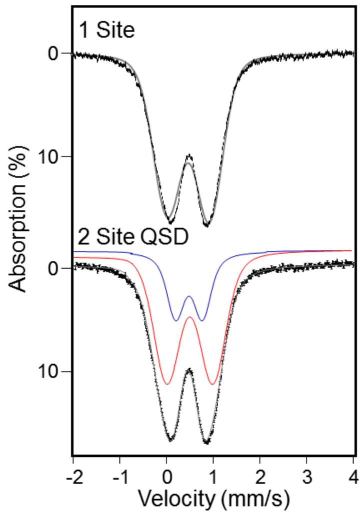

(a) (c)

Figure S9. (a) Zero-field ^{57}Fe Mössbauer spectra recorded at 80 K for the fresh sample (**1**), the sample exposed to atmosphere for 8 h, 15 h, 2 days (**1-2d**), and 180 days (**1-180d**). The simulations (solid grey lines) are obtained considering a sum of simulations obtained for high-spin ferrous (red) and ferric (blue) sites which are drawn individually above the experimental spectra, see Table S7; (b) Distribution in ΔE_Q values as determined from the simulations of the 180 days 0T, 80 K spectrum. The grey curve is the sum of the two individual components shown in red and blue. (c) Alternative simulations of the 80 K, 0 T spectrum recorded for the sample exposed to atmosphere for 180 days. To rationalize the observed spectrum we have considered both a single-site simulation (*top*) and a distribution in quadrupole splitting values (QSD) (*bottom*). The parameters obtained from this simulation are listed in Table S7.

Table S7. Hyperfine splitting parameters determined from the simulations of the 80 K, 0 T spectra presented in Figure S9a.

**Compounds**	**Oxidation State**	δ [a] **(mm/s)**	ΔE_Q [b] **(mm/s)**	Γ [c] **(mm/s)**	**A[d] (%)**
**1**	2+	1.22	3.25	0.38	100
**1-8h**	2+	1.24	3.08	-0.46	72

	3+	0.53	1.06	0.64	28
**1-15h**	2+	1.24	3.01	-0.46	67
	3+	0.55	0.98	0.64	33
**1-2d**	3+	0.44	0.97	-0.82	75
	3+	0.46	0.49	-0.39	25
**1-180d**	3+	0.44	0.97	-0.82	75
	3+	0.46	0.49	-0.39	25

[a] δ = Isomer shifts; [b] ΔE_Q = Quadrupole splitting; [c] Γ = width at half peak height and A = resonant absorption area normalized to 100% for total resonant area. The numerical values of δ, ΔE_Q and Γ were generated using WMOSS (WEB Research, Edina, MN) and are expressed in millimeter per second (mm/s).

Table S8. Hyperfine splitting parameters used for the simulations of the 80 K, 0 T spectrum of Figure S9b and S9c.

**Model**	δ (mm/s)	ΔE_Q (mm/s)	Γ (mm/s)	$d\Delta E_Q$	%
1 Site	0.46	0.88	-0.71	n. a.	100
QSD	0.46	1.00	-0.3	1.02	75
	0.46	0.59	-0.3	0.53	25

Table S9. Hyperfine splitting parameters used for the simulations. The entries to this table are colour coded according to the simulations of Figure 4c.

**Compounds**	**Site**	**Oxidation State**	**Fe(III)**	δ [a] (mm/s)	ΔE_Q [b] (mm/s)	Γ [c] (mm/s)	A[d] (%)
**1**	1	**2+**	**0 %**	**1.22**	**3.25**	**0.38**	100
**1-8h**	1	**2+**		**1.22**	**3.25**	**0.38**	29
	2	**2+**		1.22	2.94	0.39	47
	3	**3+**		0.53	1.26	0.33	12
	4	**3+**	**24 %**	0.47	0.69	0.5	9
	5	**3+**		0.45	1.24	0.47	3
**1-15h**	1	**2+**		**1.22**	**3.25**	**0.38**	14
	2	**2+**		1.22	2.94	0.39	55
	3	**3+**		0.53	1.26	0.33	12
	4	**3+**	**33 %**	**0.47**	**0.69**	**0.50**	14
	5	**3+**		**0.45**	**1.24**	**0.47**	5
**1-2d**	4	**3+**	**100 %**	**0.47**	**0.69**	**0.50**	67
	5	**3+**		**0.45**	**1.24**	**0.47**	33
**1-180d**	4	**3+**	**100 %**	**0.47**	**0.69**	**0.50**	67
	5	**3+**		**0.45**	**1.24**	**0.47**	33

[a] δ = Isomer shifts; [b] ΔE_Q = Quadrupole splitting; [c] Γ = width at half peak height and A = resonant absorption area normalized to 100% for total resonant area. The numerical values of δ, ΔE_Q and Γ were

generated using WMOSS (WEB Research, Edina, MN) and are expressed in millimeter per second (mm/s). All samples were examined at 80 K in zero magnetic fields.

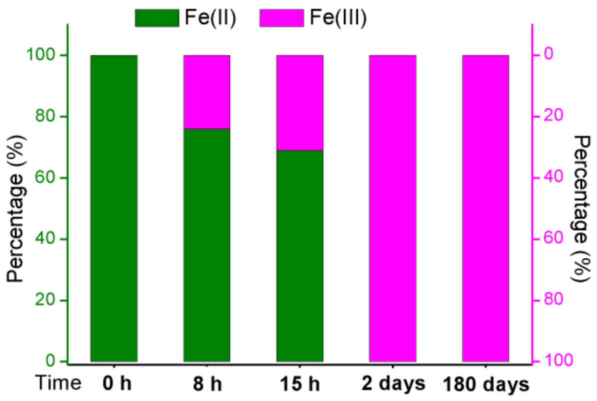

Figure S10. Relative ratio of Fe(II):Fe(III) as determined from the analyses of the Mössbauer spectra recorded for **1**, **1-8h**, **1-15h**, **1-2d** and **1-180d**.

The analysis of the Mössbauer spectra presented in Figure S9a and Table S7 allows for the successful quantification of the relative ratio of Fe(II)/Fe(III) ions. However this analysis was performed such that it considered a minimal number of independent parameters. In an effort to derive more details about the air-induced changes we have considered various similarities between the observed spectra. In order to resolve the discrepancy between the experimental and simulated spectra we have included in our simulation a distribution of ΔE_Q values (Figure S9b, Table S8). Thus Table S9 shows an alternative deconvolution, which may present a more physically meaningful interpretation of the data. For the 0 h sample we observe a quadrupole doublet characterized by parameters that are typical of high-spin ferrous ions (site 1). After 2 days of air exposure the observed spectra can be simulated only considering two ferric sites that have a 67:33 relative ratio (sites 4 and 5). Knowledge of the initial and of the final decomposition species offers us the opportunity to assess putative intermediates that might be present in samples with an intermediate air-exposure time. After 8 h a feature appears at ~1 mm/s which, upon inspection of the 180 days sample, appears to align with the right most quadrupole doublet of the final product. We then scale the simulation intensity of the final product to match the intensity of the ~1 mm/s peak in the 8 h spectrum. To fit the two outermost peaks of the spectrum we initially considered a single quadrupole doublet but found such a fit to be unsatisfactory. A more satisfying fit employs two quadrupole doublets, the first of which consists of the same parameters used for the all ferrous compounds (site 1) while the second was varied to obtain the best fit (site 2). The summation of these four sites nearly reproduces the experimental data but fails to account for all of the spectral area of the leftmost and center resonances. This discrepancy was resolved by considering an additional quadrupole doublet with parameters typical of ferric ions (site 3). The 15 h sample is simulated using the same set of parameters as the 8 h sample however with different ration of the various sites. The 2 and 180 days samples are essentially indistinguishable from each other and were simulated using a unique set of parameters. The percentages of Fe(II) and Fe(III) for each sample are list in Table S9. This alternative spectral analysis allows us to

speculate the decay process and types of products formed. We propose that upon air exposure some of the sites present in the all ferrous compound begin to oxidize yielding mixed ferrous and ferric clusters (sites 2 and 3). The appearance of the final product in the first time point suggests that the conversion once started is driven quickly, compared to the time needed to convert the entire sample, to the final state. This could be caused by a reduction in the energy barrier for subsequent oxidations or, more likely, the result of molecules on the surface shielding the unreacted bulk from oxidation.

AC susceptibility measurements

To further characterize the magnetic ground state of these two compounds, **1** and **1-2d** were also studied by AC susceptibility measurements in the temperature range of 2-18 K at zero magnetic field, but no significant out-of-phase signals were observed (Figure S11). Therefore, this compound does not behave as a single-molecule magnet in this temperature range.

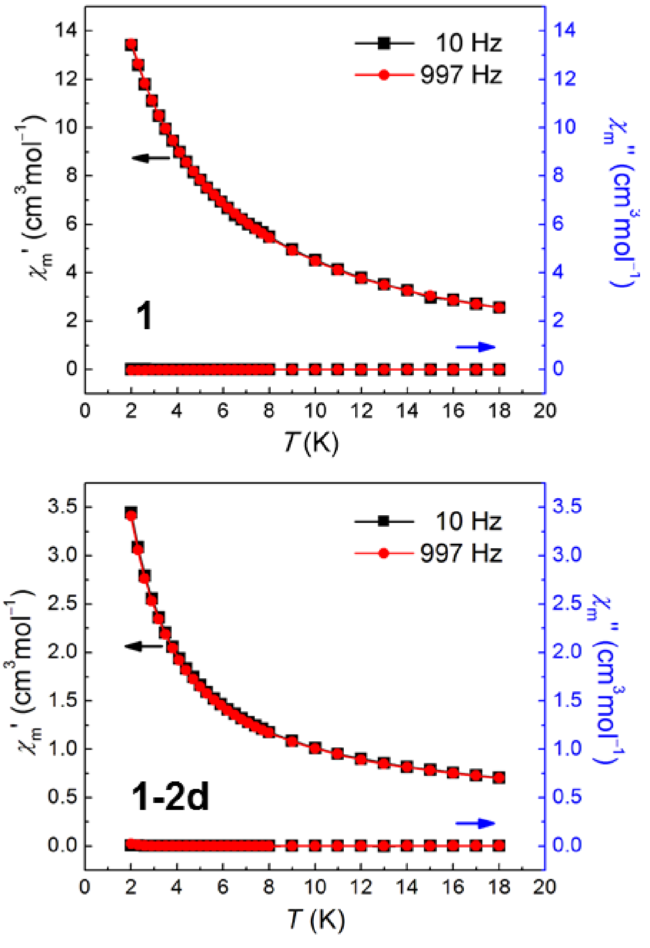

Figure S11. AC susceptibilities of **1** (top) and **1-2d** (bottom) at zero bias magnetic field.

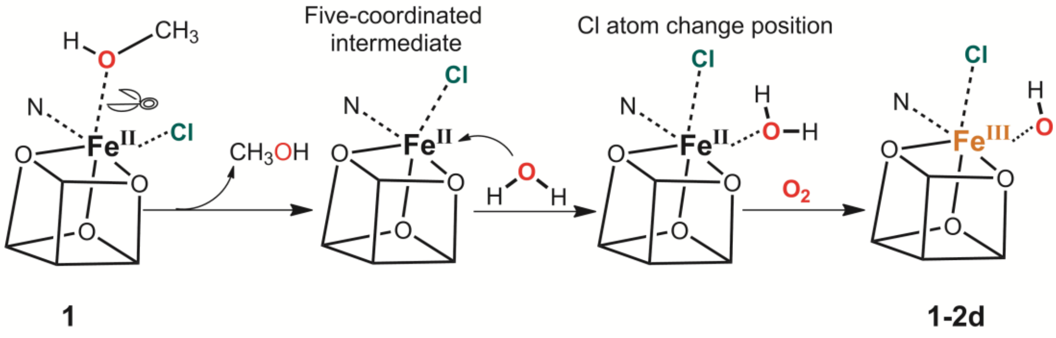

Figure S12. Schematic plausible transformation process for ligand exchange and rearrangement from **1** to **1-2d**.
